# “OPTImAL”: an ontology for patient adherence modeling in physical activity domain

**DOI:** 10.1186/s12911-019-0809-9

**Published:** 2019-04-25

**Authors:** Kristina Livitckaia, Vassilis Koutkias, Evangelia Kouidi, Mark van Gils, Nikolaos Maglaveras, Ioanna Chouvarda

**Affiliations:** 1 Lab of Computing, Medical Informatics & Biomedical Imaging Technologies, School of Medicine, Aristotle University of Thessaloniki, Thessaloniki, Greece; 2 Institute of Applied Biosciences, Centre for Research and Technology Hellas, Thermi, Thessaloniki, Greece; 3 Laboratory of Sports Medicine, School of Physical Education and Sports Sciences, Aristotle University of Thessaloniki, Thessaloniki, Greece; 4 VTT Technical Research Centre of Finland Ltd, Tampere, Finland; 5 Department of Industrial Engineering and Management Sciences, McCormick School of Engineering and Applied Science, Northwestern University, Evanston, IL USA

**Keywords:** Ontology, Knowledge base, Research data modeling, Patient adherence, Health behavior, Physical activity, Exercise, Cardiovascular disease, Rehabilitation

## Abstract

**Background:**

Maintaining physical fitness is a crucial component of the therapeutic process for patients with cardiovascular disease (CVD). Despite the known importance of being physically active, patient adherence to exercise, both in daily life and during cardiac rehabilitation (CR), is low. Patient adherence is frequently composed of numerous determinants associated with different patient aspects (e.g., psychological, clinical, etc.). Understanding the influence of such determinants is a central component of developing personalized interventions to improve or maintain patient adherence. Medical research produced evidence regarding factors affecting patients’ adherence to physical activity regimen. However, the heterogeneity of the available data is a significant challenge for knowledge reusability. Ontologies constitute one of the methods applied for efficient knowledge sharing and reuse. In this paper, we are proposing an ontology called OPTImAL, focusing on CVD patient adherence to physical activity and exercise training.

**Methods:**

OPTImAL was developed following the Ontology Development 101 methodology and refined based on the NeOn framework. First, we defined the ontology specification (i.e., purpose, scope, target users, etc.). Then, we elicited domain knowledge based on the published studies. Further, the model was conceptualized, formalized and implemented, while the developed ontology was validated for its consistency. An independent cardiologist and three CR trainers evaluated the ontology for its appropriateness and usefulness.

**Results:**

We developed a formal model that includes 142 classes, ten object properties, and 371 individuals, that describes the relations of different factors of CVD patient profile to adherence and adherence quality, as well as the associated types and dimensions of physical activity and exercise. 2637 logical axioms were constructed to comprise the overall concepts that the ontology defines. The ontology was successfully validated for its consistency and preliminary evaluated for its appropriateness and usefulness in medical practice.

**Conclusions:**

OPTImAL describes relations of 320 factors originated from 60 multidimensional aspects (e.g., social, clinical, psychological, etc.) affecting CVD patient adherence to physical activity and exercise. The formal model is evidence-based and can serve as a knowledge tool in the practice of cardiac rehabilitation experts, supporting the process of activity regimen recommendation for better patient adherence.

**Electronic supplementary material:**

The online version of this article (10.1186/s12911-019-0809-9) contains supplementary material, which is available to authorized users.

## Background

### Adherence to physical activity in patients with cardiovascular disease

Cardiovascular disease (CVD) is the leading mortality cause worldwide with an estimated 17.5 million deaths in 2012, while the number of deaths due to CVD is expected to reach 22.2 million by 2030 [1]. Many well-known factors are affecting CVD prevalence and evolution, such aging, gender, lifestyle behavior (e.g., tobacco use, excessive alcohol consumption, insufficient level of physical activity), etc. [1].

Physical fitness is a significant factor related to CVD management and a crucial component of the therapeutic process for patients with heart disease. In particular, an increase in physical activity levels over time has been associated with reduced coronary heart disease (CHD), and CVD mortality risk in general [2]. Moreover, exercise-based cardiac rehabilitation programs (CRPs) followed after revascularization or myocardial infarction are associated with a mortality reduction in the range of 20 to 30% [3]. Despite the recognized importance of being physically active, patient adherence to exercise, both in daily life and during cardiac rehabilitation, is low. Even patients who participate in CRPs of different duration, show a low level of adherence with drop-out rates up to 56% [3]. The term “adherence” is defined by the World Health Organization (WHO) as “the extent to which a person’s behaviour – taking medication, following a diet, and/or executing lifestyle changes, corresponds with agreed recommendations from a health care provider” [4].

Factors from various perspectives are influencing patient adherence, including social, economic, healthcare system, physiological, therapy-related, lifestyle, etc. For example, older age or low level of education results in a poorer adherence to cardiac rehabilitation [3]. Similarly, the presence of depression, anxiety, and insufficient social support might reduce adherence to physical activity [3]. Regarding lifestyle-related choices, some patients may prefer home-related activities, such as gardening, instead of following a specific recommendation for exercising or joining a CRP [3, 5]. Moreover, not all hospitals and rehabilitation facilities have programs for particular types of patients, e.g., those suffering from heart failure, while the number of symptoms (e.g., fatigue, experiencing angina) associated with this condition might be a barrier itself in participating in existing CRPs [3].

### Physical activity recommendations and interventions in healthcare

Changing CVD behavioral risk factors (e.g., modifiable factors such as motivation, enjoyment, etc.) with specific interventions is estimated to contribute to a decrease in CVD mortality in high-income countries as much as 50% [6]. Despite the recognized problem of poor adherence to physical activity and exercise training, there is still not enough scientific evidence on successful interventions’ design and potential impact, targeting adherence improvement or maintenance [3, 5, 7].

In 2015, 67% of the countries participating in the WHO survey reported the establishment of evidence-based national guidelines, protocols, and standards, developed for use by health professionals for the management of CVD [8]. However, only 53% of the countries having guidelines reported their full implementation [8]. In many cases, services in primary health care are either unavailable or unstructured and not always following evidence-based practices [1]. As there are no primarily-recommended strategies to improve adherence, healthcare professionals are expected to use their skills, available resources, and patient preferences to adjust methods for enhancing patient adherence to physical activity regimen [1]. In most cases, specialists working with CVD patients are not trained to provide personalized counseling, and the advice to a patient is conveyed in a rather general way as to the necessary actions required by the patient [3]. The practice of medical doctors working with patients shows that the available information that might clarify the situation with the patient or adjust an intervention is accessed through bibliographic databases (e.g., MEDLINE) [9–11]. However, this process is cumbersome and cannot be efficiently implemented or reused in practice.

### Research data reusability through ontologies and related work

Knowledge regarding patient adherence to physical activity may support the design of successful strategies for recommendations and interventions [3]. However, the heterogeneity of data available from the conducted studies regarding patient populations, study contexts, and description of study results is a significant challenge for researchers in reusability of available data results [12]. Ontologies constitute one of the methods applied for efficient knowledge sharing and reuse [13]. An ontology allows combining conceptual knowledge with quantitative and qualitative data, supporting interoperability and flexibility of its underlying model [14].

To provide an overview of existing formal models related to the domain of our interest, we explored scientific databases and ontology repositories for existing models using the keywords “adherence ontology,” “physical activity adherence ontology,” and “exercise adherence ontology.” In particular, we searched MEDLINE and IEEE Xplore [15]. The ontology repositories searched included BioPortal [16], the Open Biological and Biomedical Ontology (OBO) Foundry [17], and the Ontology Lookup Service (OLS) [18]. We analyzed two additional ontological resources found separately [19, 20]. We then reviewed the purpose and domain of the ontologies to consider their relevance to our work and potential reuse. The identified, related ontological resources are listed in Table 1.

**Table 1:** Review of physical activity and exercise-related ontologies

Resource	Purpose of the ontological resource	Domain
Kostopoulos et al. [21], 2011	To support personalized exercise prescription	Exercise in cardiac rehabilitation
Faiz et al. [22], 2014	To recommend diet and exercise based on the user profile	Diet and exercise in diabetes patients
Foust [23], 2013	To provide a reference for describing an exercise regarding functional movements, engaged musculoskeletal system parts, related equipment or monitoring devices, and intended health outcomes	Anatomy of exercise and health outcomes
Bickmore & Schulman [19], 2013	To describe health behavior change interventions (exercise and diet promotion)	Health behavior change (exercise and diet)
Colantonio et al. [20], 2007	To model the domain knowledge base and represent formalism, knowledge sharing, and reuse	Heart failure patient clinical profile

We found of particular interest, the ontology-based framework developed for personalized exercise prescription for patients with heart disease by Kostopoulos et al. [21]. The framework combines medical domain-related knowledge and inference logic to propose exercise plans for each patient as a decision support tool for healthcare professionals. The personalization of the framework is based on the patient’s preferences for exercise types (e.g., cycling, jogging), the time of the day for planned activity, and lifestyle aspects (e.g., previously sedentary lifestyle). The study associated an improved adherence with a positive attitude and acceptance of a prescribed plan by a patient. As a part of personalization inference, no more factors influencing patient adherence were included. Two other ontologies supported personalized exercise recommendation based on the patient’s profile [22, 23]; however, the concept of adherence to physical fitness was not addressed. The ontology promoting health behavior change in the domain of exercise did not include concepts related to adherence behavior [19]. The ontological resource in heart failure domain was found to be a comprehensive representation of a clinical patient-related perspective but lacking in the relevance of patient adherence in the physical activity domain [20].

Despite that all the reviewed ontologies helped us to develop our original approach for the ontology design, none of them supported the combination of a multidimensional patient profile (e.g., personal, environmental, clinical, and other factors) and patient adherence to physical activity-related behavior, or other concepts that could support the interpretation of the relationships between the adherence and patient-related factors. Therefore, we found it necessary to develop a new ontology that will incorporate patient profile and its association with adherence to physical activity regimen in the domain of CVD patient adherence to physical activity and exercise.

### Rationale, our aim and contribution to the field

Despite the fact that the therapeutic exercise training is studied for decades from the perspective of several domains (sports medicine, cardiology, physiology, etc.), comprehension of individualized patient adherence remains insufficient [24]. Research in the domain produced a large body of evidence regarding factors affecting CVD patients’ adherence to physical activity regimen. However, most of the results are partial and majorly differ in the specific characteristics in CVD patient population (e.g., gender, comorbidity, race, etc.). Moreover, often the aspects of adherence and physical activity and fitness are not considered as a valuable component in studying the adherence concept. This approach considerably influences the recognition of factors affecting particular adherence to a specific activity component and, as a result, the research evidence might not be used or be misinterpreted in the medical practice. Therefore, there is a need for a semantic foundation that allows organization of available research data into domain-specific ontology targeting relations of factors affecting CVD patients’ adherence.

In this study, we introduce OPTImAL, a reusable ontology model focusing on physical-activity-related adherence of patients with heart disease. Through OPTImAL, the relations of different factors to adherence and adherence quality, as well as the associated types and dimensions of physical activity and exercise training are described. Our contribution to the field concerns the introduction of a comprehensive approach exploiting knowledge available in the scientific literature and organizing the results into a Knowledge Base (KB) to be used as part of decision support in the medical practice of experts targeting CVD patients’ adherence with physical activity regimen.

## Methods

**Fig. 1:**
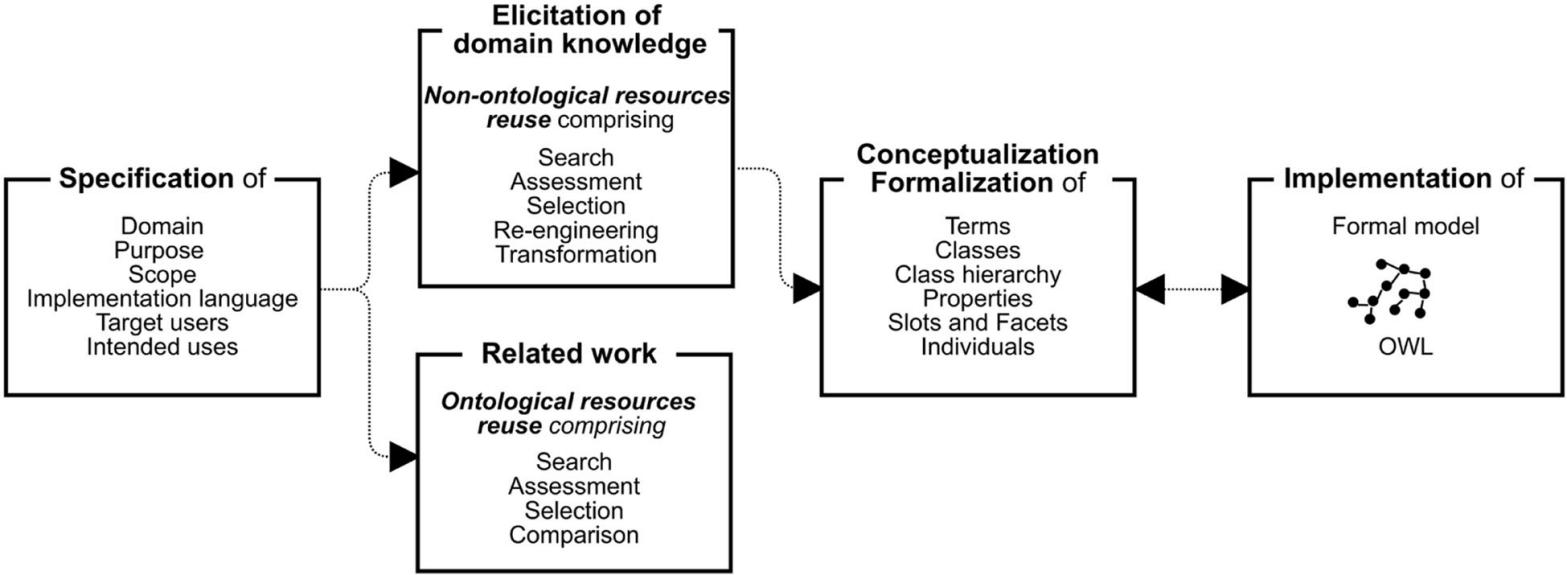
Ontology development process

OPTImAL was developed following the Ontology Development 101 methodology [25], and refined based on the NeOn framework [26]. The implementation decision was made based on the essential requirement for combining: (a) ontology design methods (ontology specification and elicitation of knowledge), and (b) ontology design principles (established by the Ontology Development 101). OPTImAL was developed following the process presented in Fig. 1. Further, OPTImAL was validated for its consistency through automatic reasoners, while a preliminary assessment conducted by a cardiologist and three CR trainers evaluated OPTImAL regarding its appropriateness and usefulness in the domain.

### Ontology development

#### Ontology specifications

Considering physical fitness as one of the essential recommendations for patients with CVD, we narrowed the scope of OPTImAL to physical activity and exercise adherence in patients with heart disease. Further, a list of competency questions that should be answered using OPTImAL as a support tool was compiled. Most of the documented questions were as follows: “*What factors are associated with adherence to daily physical activity?*”; “*What determinants are motivating the patient to adhere to long-term outpatient cardiac rehabilitation program?*”; “*What are the barriers to exercise in different settings for female/male patient population?*”. In Table 2, we describe the OPTImAL specifications concerning its purpose, scope, implementation language, target users, and intended use (i.e., as the knowledge component of a decision support system), adapted from [26].

**Table 2:** OPTImAL specifications

Purpose	The purpose of the ontology is to express factors related with adherence to physical activity or exercise training, characterizing the patient profile (e.g., demographics, lifestyle, social support, physiological condition, etc.).
Scope	The ontology should focus on physical-activity-related adherence of patients with heart disease.
Implementation language	Web Ontology Language (OWL)
Target users	The primary target users are healthcare professionals working with cardiac patients, aiming to recommend (User 1) or coach them (User 2) on physical activity and exercise. Another group of target users (User 3) is professionals involved in the development of software solutions to support physical activity and exercise performance of patients, i.e.: User 1 – Cardiologist User 2 – Cardiac rehabilitation program trainer User 3 – Software developer or researcher working in the domain related to CVD patient adherence in physical activity domain
Intended uses	User 1: The intended uses of the ontology include: (1) supporting the process of physical activity regimen recommendation to a patient and (2) modifying current physical activity regimen for better adherence. User 2: The intended use is to support the process of modification of patient adherence based on changes in the patient profile. User 3: The intended use is to support the development of personalized solutions for physical activity maintenance and modification based on the patient’s need.

#### Elicitation of domain knowledge

For defining OPTImAL, we primarily considered the outcomes of the literature review conducted within the Connected Health Early Stage Researcher Support System (CHESS) project; under the framework of Marie Skłodowska-Curie grant agreement (676201) [27]. The literature review aimed to comprehensively examine published literature addressing factors associated with physical activity behavior adherence in patients with heart disease. The results of the review outline associated factors and their complex relations to physical-activity-related adherence, which may allow optimizing patient assessment approaches, intervention strategies, and recommendations tailoring for activity adherence support and modification. To verify the results, we iterated the review following the methodology described in [27]. We then extended the results of the analysis concerning the specifications of the ontology. Based on the literature analysis, we outlined patient-related factors (e.g., age, motivation, lifestyle, etc.) associated with a particular type of adherence, its quality under a specific kind of physical fitness and settings (e.g., independent physical activity, an exercise in CRP, etc.). In the analysis, we included factors that were studied but not significantly associated with patient adherence. The ontology is based on 41 published studies. The results of the analysis are summarized in Additional files 1, 2 and 3. Part of the extended review targeting types and settings of physical activity as well as exercise behavior associated with adherence has been previously published [28].

#### Conceptualization and formalization

After selecting and refining the core knowledge that would be incorporated into the proposed ontology, we proceeded with non-ontological resources re-engineering to transform the selected domain knowledge into the ontology concepts. First, we identified the general terms of our ontology, including:

- factors associated with patient adherence (patient profile),
- relations of factors to adherence,
- types and settings of adherence behavior, and
- types and context of physical activity behavior associated with adherence.

Then, the extracted terms were enumerated into concepts (classes) using combination development process [25]. To define the class hierarchy, we started with high-level concepts (e.g., ActivityAdherenceType) and then detailed them (e.g., Adherence, Attendance, Completion, etc.). We followed a bottom-up development process, grouping specific concepts (e.g., PhysicalActivity, Exercise, CardiacRehabilitationExercise, etc.) into classes representing more general concepts (e.g., ActivityBehavior). The relations among concepts were defined through object properties. In our development, part of the identified terms was defined as object properties (e.g., the term “relations of factors to adherence” corresponds to the property hasRelationType of class Factor). For each object property, we assigned the class that it describes. Object properties were defined with facets, representing features of the values the property can take (i.e., cardinality, value type, and domain and range). As the last step before implementing the ontology, we created a list of individuals and organized them into themes of the patient profile factors. For example, an individual of type DepressionScore was classified under the class DepressionFactor.

#### Implementation

OPTImAL was implemented in the OWL2 language using Protégé Desktop (version 5.2.0) [29]. During the process of development, we applied visualization plug-ins such as VOWL [30] and OntoGraf [31], as well as the OAF plugin for deriving the ontology abstraction network [32], i.e., an algorithmically-derived summary of an ontology’s structure and content.

### Ontology validation and evaluation

#### Validation of the ontology consistency

To validate the consistency of OPTImAL, we applied HermiT (version 1.3.8.413), a reasoner incorporated in Protégé. HermiT is a reasoner for OWL ontologies that can determine whether or not the ontology is consistent [33]. In particular, the reasoner was set to check class, object property, and individual inferences. The ontology was validated for its logical consistency as a necessary procedure that has to be accomplished to continue with preliminary evaluation.

#### Evaluation of appropriateness and usefulness of the ontology

After validating the ontology for consistency, we proceeded in its evaluation of appropriateness and usefulness in medical practice, so that OPTImAL can be employed in planning activity regimen recommendation and modification for a patient. Before the ontology was reviewed by the domain expert (cardiologist), an introductory session took place. In the session, the concept of ontologies has been explained to the cardiologist along with illustrative examples from the biomedical domain. The expert was asked to give four questions of daily practice. The proposals were: “*What are the reasons for non-adherence to a 1-year-duration cardiac rehabilitation program for women?*”; “*Are there any patient determinants that facilitate adherence to long-term outpatient cardiac rehabilitation?*”; “*What adherence-related factors studied/found in a group of patients with heart disease and depression?*”, and “*What are predictors of adherence to exercise after index hospitalization?*”. Using the DL (description logic) Query tab of Protégé, each question we interpreted as class expression (query) that was recognized by the reasoner HermiT. The interface of the tab allowed to search the classified ontology, test the syntax, and evaluate the query results [34]. To find the reasons for non-adherence to a 1-year-duration cardiac rehabilitation program in women, we constructed Query 1. To identify patient determinants that facilitate adherence to long-term outpatient cardiac rehabilitation, we created Query 2. To retrieve studied factors related to adherence in patients with heart disease who have depression, we formed Query 3. Query 4 was constructed to identify predictors of adherence to exercise after index hospitalization. Once the results of the queries were available, the cardiologist assessed if they could potentially be applied in the decision making for a patient recommendation.

Additionally, we asked the cardiologist to reproduce the scenario when a patient comes for a visit, as a case study. The cardiologist has described the following: “Patient participates in outpatient cardiac rehabilitation program organized by the municipality. This patient has a depression, and during CRP, motivational techniques were used to maintain the program participation. The CRP will be temporally terminated for four months period. It is critical to understand what could facilitate the adherence before the CRP continuation, so can be applied to intervention planning.” Based on the case, the question was formed as follows “What are the facilitators of adherence to physical activity after a cardiac rehabilitation program in a group of patients with depression?” To retrieve the factors facilitating adherence to physical activity after completing a cardiac rehabilitation program in a patient with depression, we constructed Query 5. Table 3 comprises the evaluation questions and the case study question and the corresponding class expressions.

**Table 3:** Evaluation questions and corresponding class expressions

“What are the reasons for non-adherence to a 1-year-duration cardiac rehabilitation program for women?”	
Query 1:	hasRelationTo some NonAdherenceToCrUp1YearPeriod and (hasRelationType value Reason) and (isStudiedIn value FemalePopulation)
“Are there any patient determinants that facilitate adherence to long-term outpatient cardiac rehabilitation?”	
Query 2:	hasRelationTo some AdherenceToCrMore3YearsPeriod and (hasRelationType value Facilitator)
“What adherence-related factors were studied in a group of patients with heart disease and depression?”	
Query 3:	isStudiedIn value DepressionPopulation
“What are predictors of adherence to exercise after index hospitalization?”	
Query 4:	hasRelationTo some AdherenceToExerciseAfterIh and (hasRelationType value Predictor)
“What are the facilitators of adherence to physical activity after a cardiac rehabilitation program in a group of patients with depression?”	
Query 5:	hasRelationTo some AdherenceToPaAfterCr and (hasRelationType value Facilitator) and (isStudiedIn value DepressionPopulation)

Three CR trainers were also asked to answer two open questions regarding the ontology appropriateness (if the ontology suits daily practice) and usefulness (if the ontology supports CR practice). The questions are: “*Do you see the demonstrated ontology as a suitable tool in your daily practice in cardiac rehabilitation?*”; “*Do you believe that the demonstrated ontology could support your daily practice in cardiac rehabilitation?*”

## Results

### Description of the developed ontology

#### Ontology metrics

OPTImAL includes 142 classes, ten object properties, and 371 individuals that describe relations of CVD patient profile factors with adherence to physical activity and exercise. To comprise the overall concepts that the ontology describes in its application domain, we defined 2637 logical axioms. Regarding the kinds of axioms and the kinds of class expressions used in OPTImAL, the description logic was expressed through ALCOI (the base attribute language AL followed by extension to refer to complex concept negation, individual objects (nominals), and inverse properties). The main ontology metrics are summarized in Table 4, providing the extent of the underlying model and its elements.

**Table 4:** Ontology metrics

Metrics	
Axioms	3170
Logical axiom count	2637
Class count	142
Object property count	10
Individual count	371
DL expressivity	ALCOI
Class axioms	
SubClassOf	72
EquivalentClasses	67
DisjointClasses	12
Object property axioms	
ObjectPropertyDomain	10
ObjectPropertyRange	10
Individual axioms	
ClassAssertion	877
SameIndividual	33
DifferentIndividuals	1554

#### Classes and class hierarchy

**Fig. 2:**
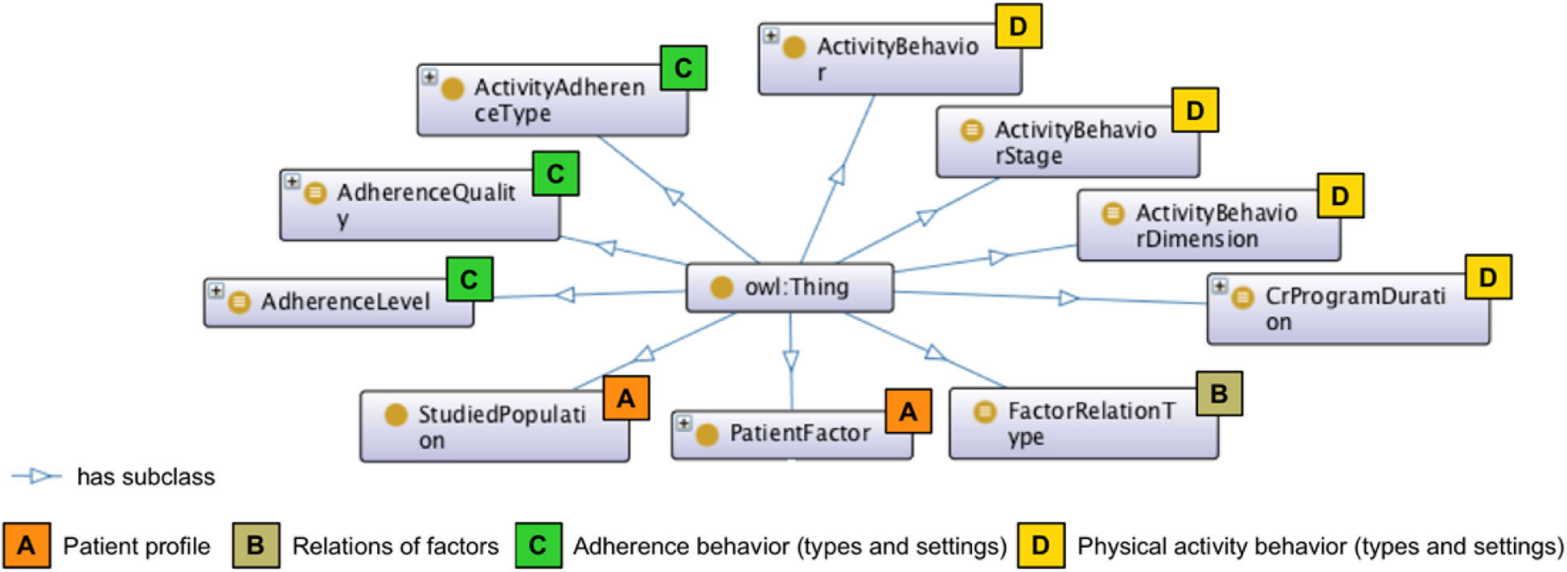
Top-level classes and ontology domain concepts

The top-level classes in our formal model reflect the terms and concepts stated earlier (see subsection Conceptualization and formalization). Top-level classes consist of the classes representing patient factor, studied population, factor relation type, activity adherence type, adherence quality, adherence level, activity behavior, activity behavior dimension, activity behavior stage, and CRP duration. Figure 2 visualizes the top-level classes and their relation to the ontology domain concepts. Classes of a patient factor, activity behavior, and activity adherence type are divided into subclasses. The rest of the top-level classes consist of individuals for concepts description, and in most cases, represent equivalence of individuals as described further (see subsection *Individuals*). In OPTImAL, top-level classes are not disjoint due to their intersection and complex relations. However, to avoid ambiguity in the classification of patient profile and patient activity behavior, all subclasses of all top-level classes were disjointed, so that individuals of one class cannot be instances of more than one class in the set of subclasses.

The PatientFactor class corresponds to factors examined on their influence on adherence of CVD patients to physical activity and exercise. The identified determinants were divided into 60 subclasses based on their relation to different aspects of the patient’s life (i.e., patient demographics, anthropometrics, physiology, physical health, etc.). The class hierarchy representing the patient profile is shown in Additional file 4. Although all factors employed in the ontology were studied in the population of patients with heart disease, in several studies, the target population characteristics differed significantly (i.e., patients with CVD and depression, female CVD population, etc.). We found it to be relevant to capture particular patient characteristics into studied population class StudiedPopulation, which mainly supports an additional description of the patient profile in our ontology.

Class ActivityBehavior is divided into four subclasses that indicates a patient behavior such as physical activity, exercise, cardiac rehabilitation exercise, and activity adherence, totally summarizing eight classes of ontology. Types of activity adherence behavior, including (non-)adherence, attendance, (non-)completion, dropout, and nonparticipation, are classified under class ActivityAdherenceType. Further, we defined new classes corresponding to activity behavior dimensions, stages, levels, quality, and CR program duration (i.e., ActivityBehaviorDimension, ActivityBehaviorStage, AdherenceLevel, AdherenceQuality, CrProgramDuration, respectively). Further, indicating a combination of a specific patient activity behavior and adherence type and its settings, we implemented classes equivalent to an intersection of interrelated classes.

**Fig. 3:**
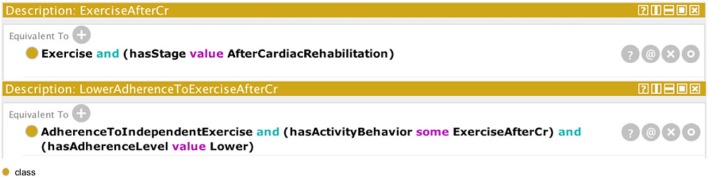
Example of classes design in Protégé

As shown in Fig. 3, to create a class representing exercise behavior after cardiac rehabilitation, we designed a class representing activity behavior (Exercise) and an individual of the class, describing the stage of the activity behavior (AfterCardiacRehabilitation). Following the same design pattern, LowerAdherenceToExerciseAfterCr class was created through the classes representing adherence to independent exercise (AdherenceToIndependentExercise) and exercise after CR (ExerciseAfterCr), and the individual of adherence level class (Lower) as illustrated in Fig. 3.

#### Object properties

**Fig. 4:**
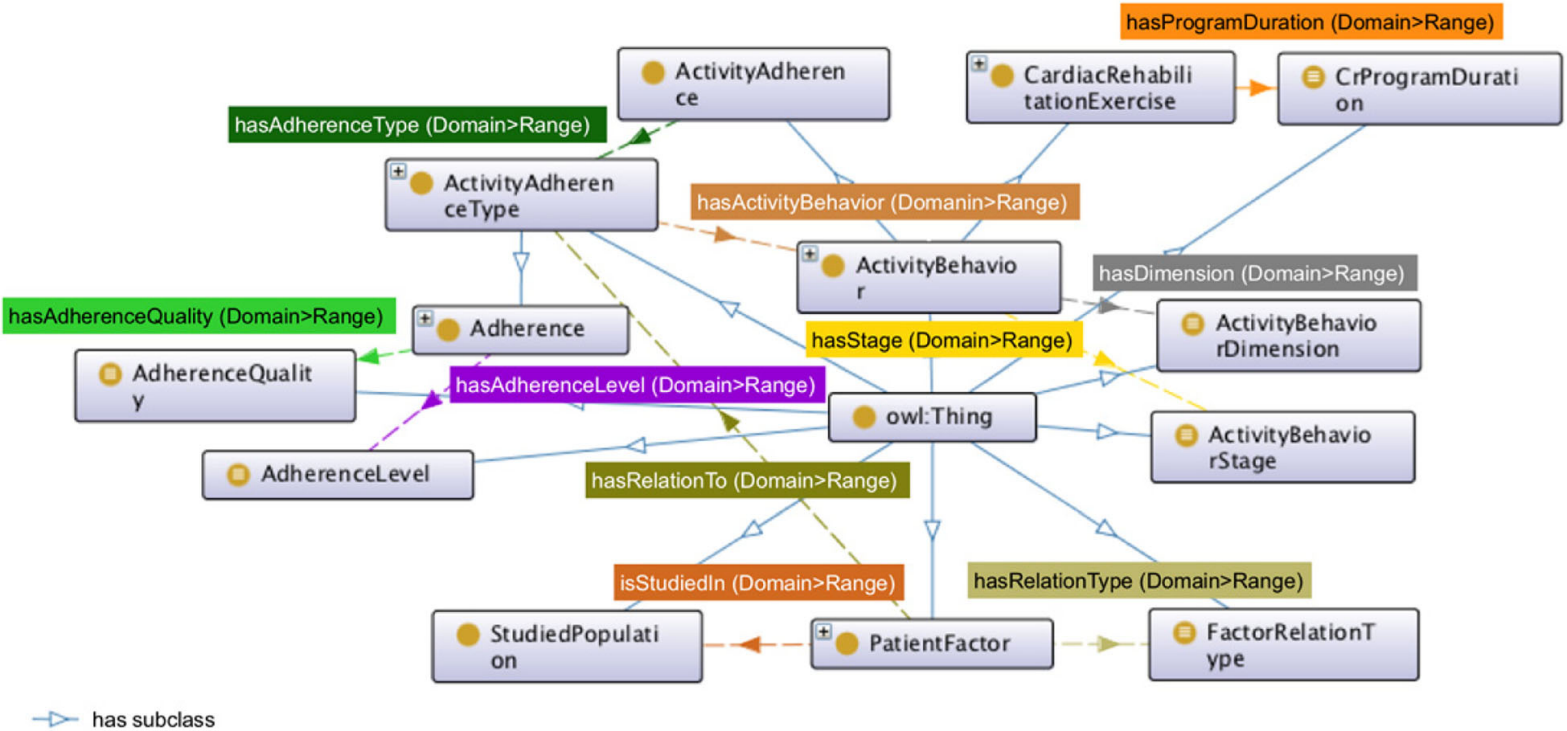
Object properties

We defined ten object properties and their facets expressing the relations of the classes described in the previous subsection (see subsection *Classes and class hierarchy*). The relations shown through the properties are: hasActivityBehavior, hasAdherenceLevel, hasAdherenceQuality, hasAdherenceType, hasDimension, hasProgramDuration, hasRelationTo, hasRelationType, hasStage, and isStudiedIn. We defined the symmetric characteristic of the hasRelationTo property. Facets of each object property were defined with domains and ranges as shown in Fig. 4.

The outlined summary of the ontology properties is based on the abstraction network (see Table 5) [32]. Twenty five classes in activity adherence class are described with the hasActivityBehavior property. Properties hasStage and hasDimension describe eight classes in ActivityBehavior class; together with hasStage property, describe ActivityAdherence class. Conjointly, properties hasRelationType, hasRelationTo, and isStudiedIn describe 60 classes in patient factor class. Five classes in cardiac rehabilitation exercise class are described by hasDimension, hasProgramDuration, and hasStage properties. Finally, properties hasActivityBehavior, hasAdherenceLevel, and hasAdherenceQuality describe 35 classes in adherence class.

**Table 5:** Summary of properties derived from ontology abstraction network

Describing property	Described class	Number of described subclasses in the class
hasActivityBehavior	ActivityAdherence	25
hasStage hasDimension	ActivityBehavior	8
hasActivityBehavior hasAdherenceLevel hasAdherenceQuality	Adherence	35
hasStage hasDimension hasStage	ActivityAdherence	1
hasRelationType hasRelationTo isStudiedIn	PatientFactor	60
hasDimension hasProgramDuration hasStage	CardiacRehabilitationExercise	5

#### Ontology individuals

**Fig. 5:**
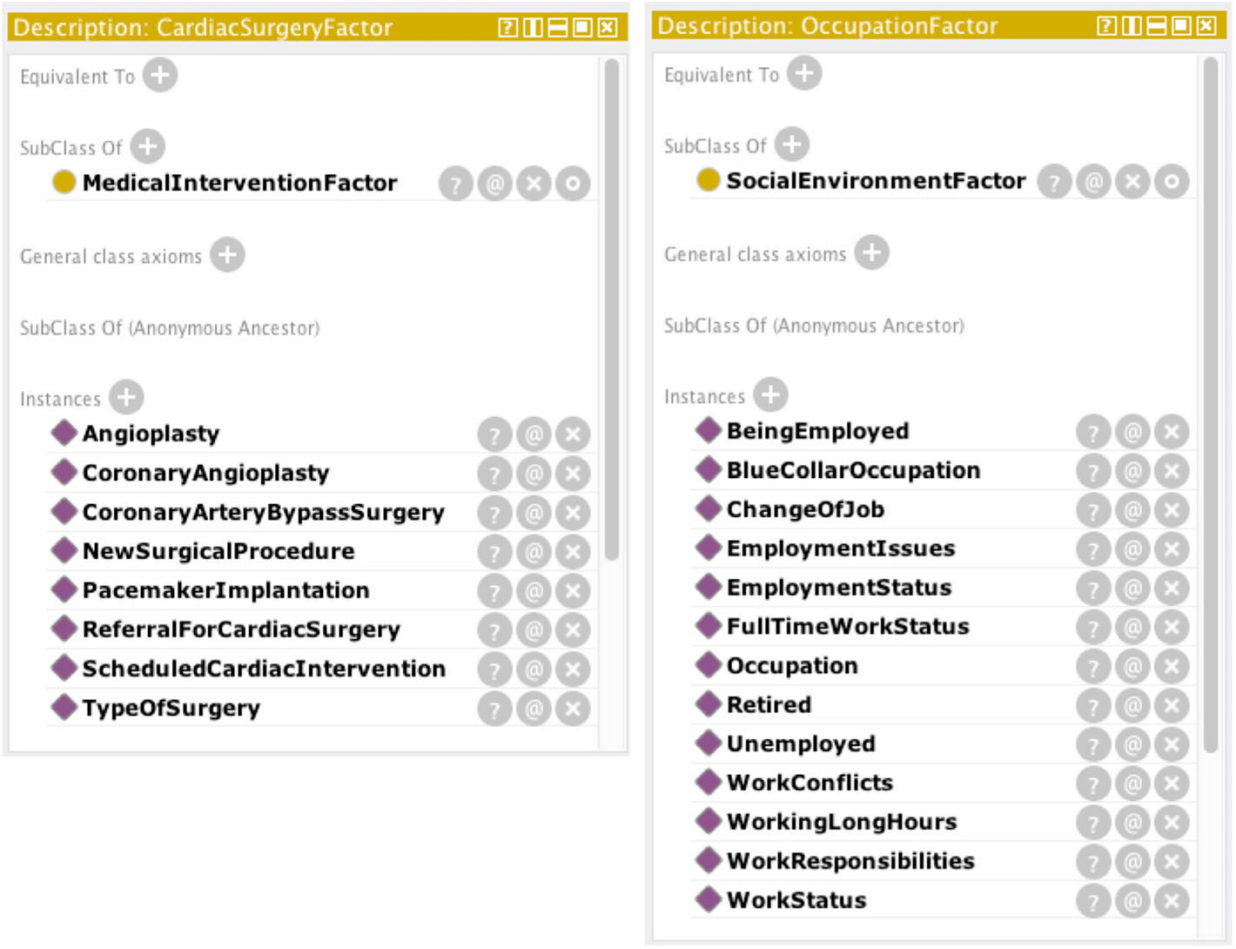
Individuals of patient factor subclasses. Example from Protégé

We defined 371 ontology individuals under eight top-level classes. Classification of individuals in PatientFactor class was held concerning different patient aspects. We divided 320 discovered factors regarding patient demographics, anthropometrics, physiology and physical health, cardiovascular disease and comorbidity, lab tests, symptoms, cognitive and psychological state, environment, social environment and social support, health behavior, health literacy, lifestyle, healthcare service, insurance, and exercise physiology and exercise settings. Individuals associated with patient profile were classified of PatientFactor class. An example of classified subclasses and associating individuals is shown in Fig. 5. Additional file 5 presents the full list of instances regarding a specific patient aspect.

**Fig. 6:**
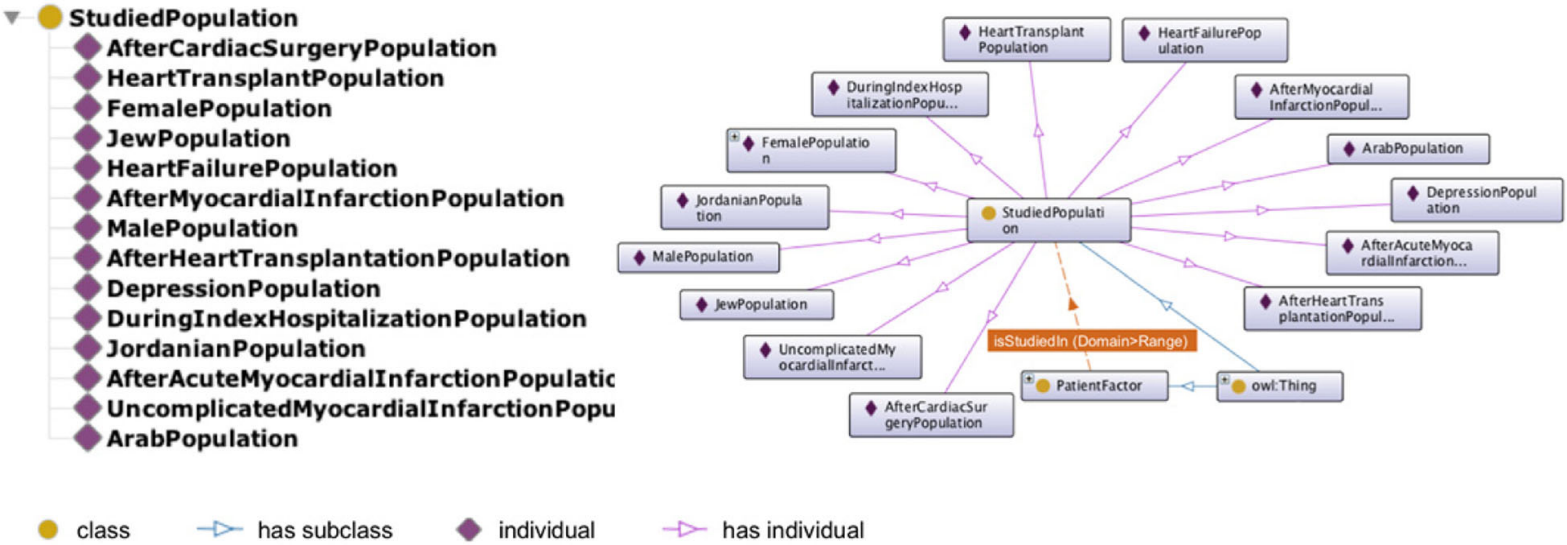
Instances of StudiedPopulation and relation with associated classes

We included 14 instances illustrating a particular characteristic of the patient population in the Studied Population class. The respective individuals include patients with depression; female and male population; Jew, Arab, and Jordanian population; study participants after cardiac surgery, heart transplantation, and (acute) myocardial infarction; patients with a history of heart failure and uncomplicated myocardial infarction, and patients with heart transplants. Figure 6 lists the individuals in StudiedPopulation class and shows a relation between StudiedPopulation and PatientFactor classes.

**Fig. 7:**
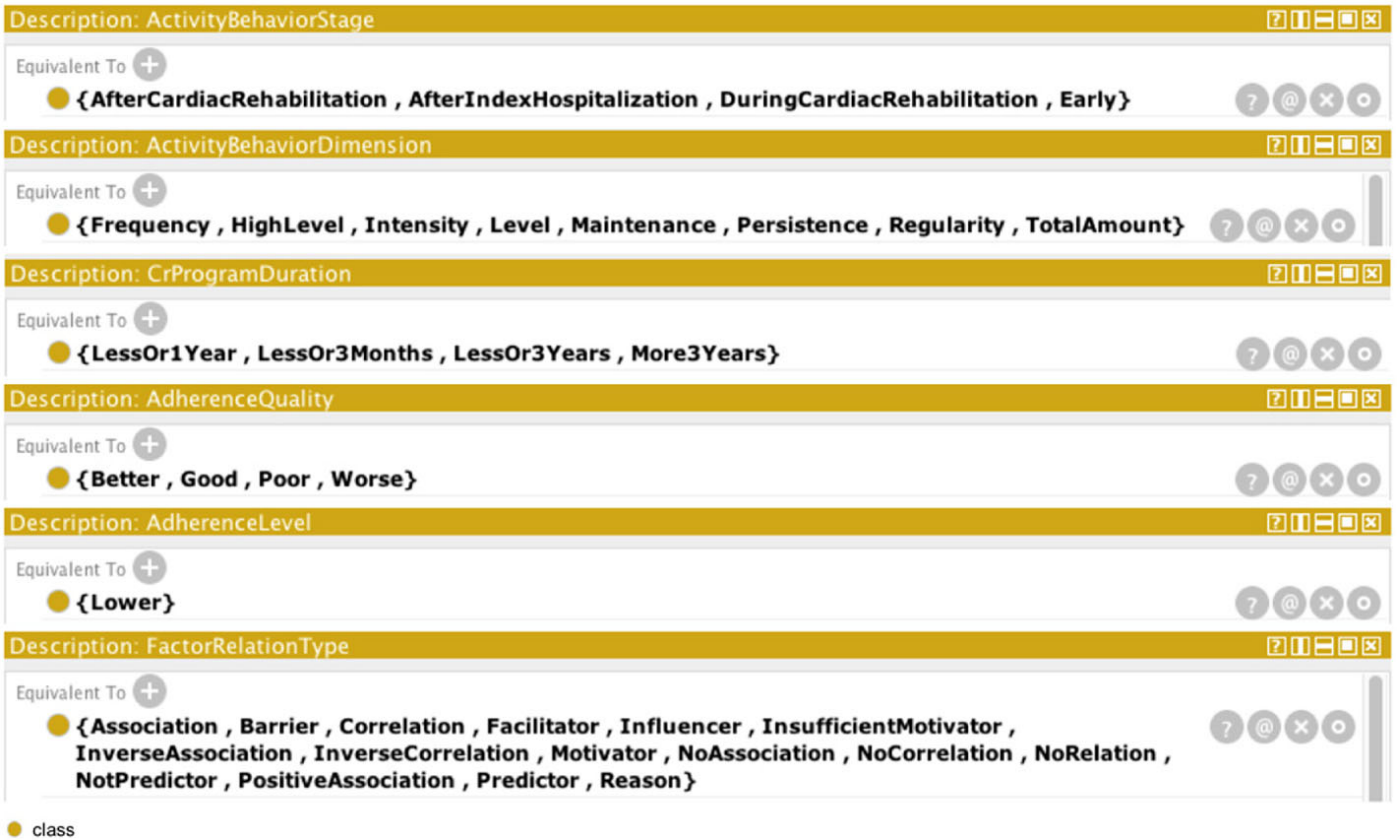
Description of classes representing activity behavior in Protégé

Classes representing activity behavior settings (i.e., stage, dimension, etc.) and types of relations between factors and adherence is an enumeration of individuals as shown in Fig. 7. Class ActivityBehaviorStage represents stages of activity behavior, and includes individuals such as early stage, after and during cardiac rehabilitation, and after index hospitalization stage. Frequency, level, intensity, maintenance, persistence, regularity, and total amount are individuals of ActivityBehaviorDimension class. CrProgramDuration class is described with individuals characterizing duration of a cardiac rehabilitation program for less or three months, less or one year, less than three years, or more than three years. Individuals describing quality settings, including better, good, poor and worse were placed in the AdherenceQuality class. Class AdherenceLevel contains only one individual, expressing a low level of adherence. We grouped in the FactorRelationType class the types of relations that are describing associations between factors and patient adherence. The relations include reason, barrier, facilitator, (insufficient) motivator, influencer, (not) predictor, (positive/inverse/no) association, (inverse/no) correlation, and no relation.

### Results of OPTImAL validation and evaluation

#### Consistency validation

The logical consistency of OPTImAL was successfully validated through the HermiT reasoner incorporated in Protégé. HermiT did not return any inconsistency regarding classes, object properties and individual inferences of the ontology.

#### Appropriateness evaluation

The independent cardiologist who evaluated OPTImAL as regards its appropriateness in the daily CR practice found that it fits the doctor’s activities involving intervention planning (including CRP) and supporting comprehension of a patient profile. As regards the usefulness of OPTImAL in the medical practice, the cardiologist proposed four (typical for the domain) questions to be answered using OPTImAL as a decision support tool (see section Ontology validation and evaluation).

**Fig. 8:**
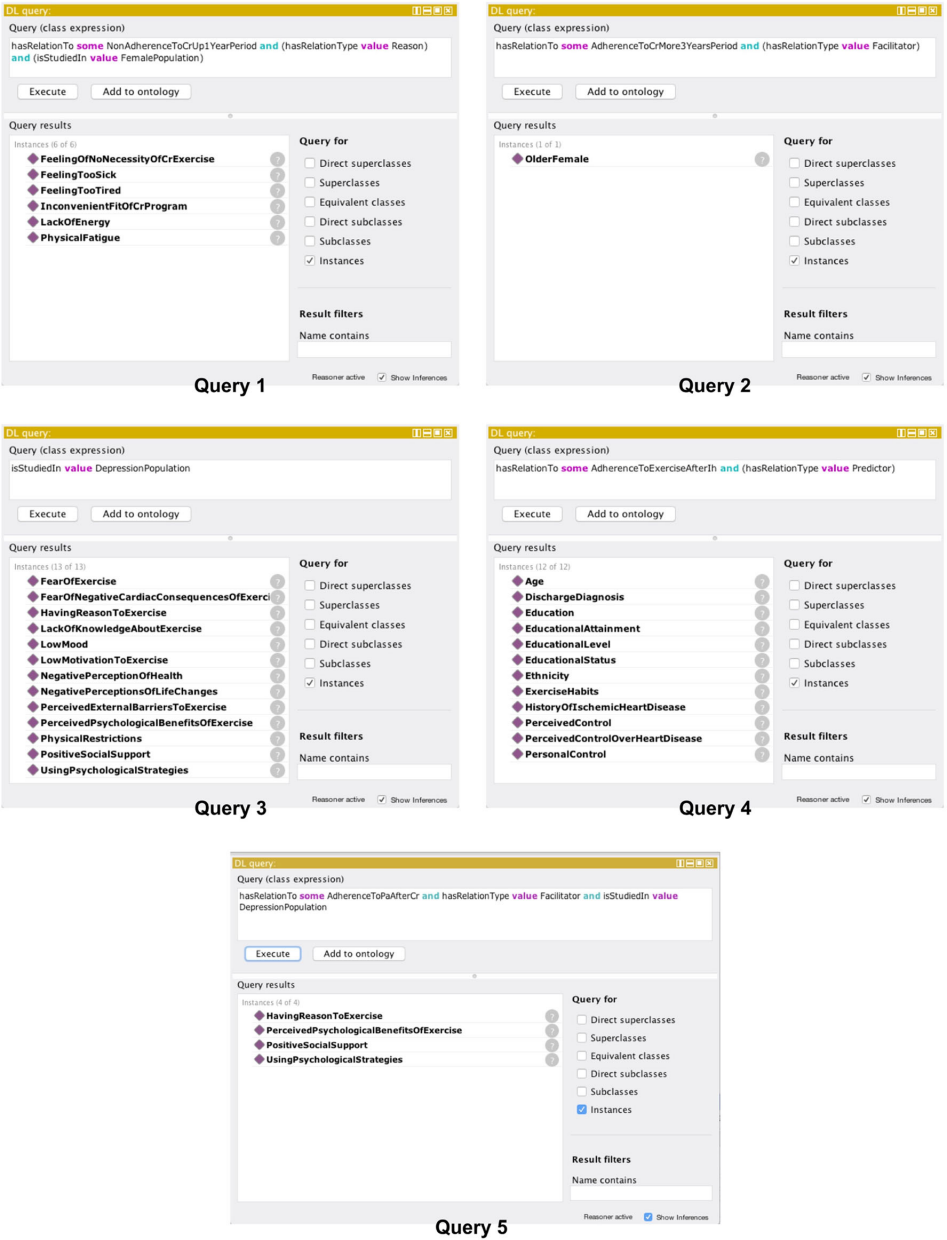
Evaluation query results in Protégé

**Fig. 9:**
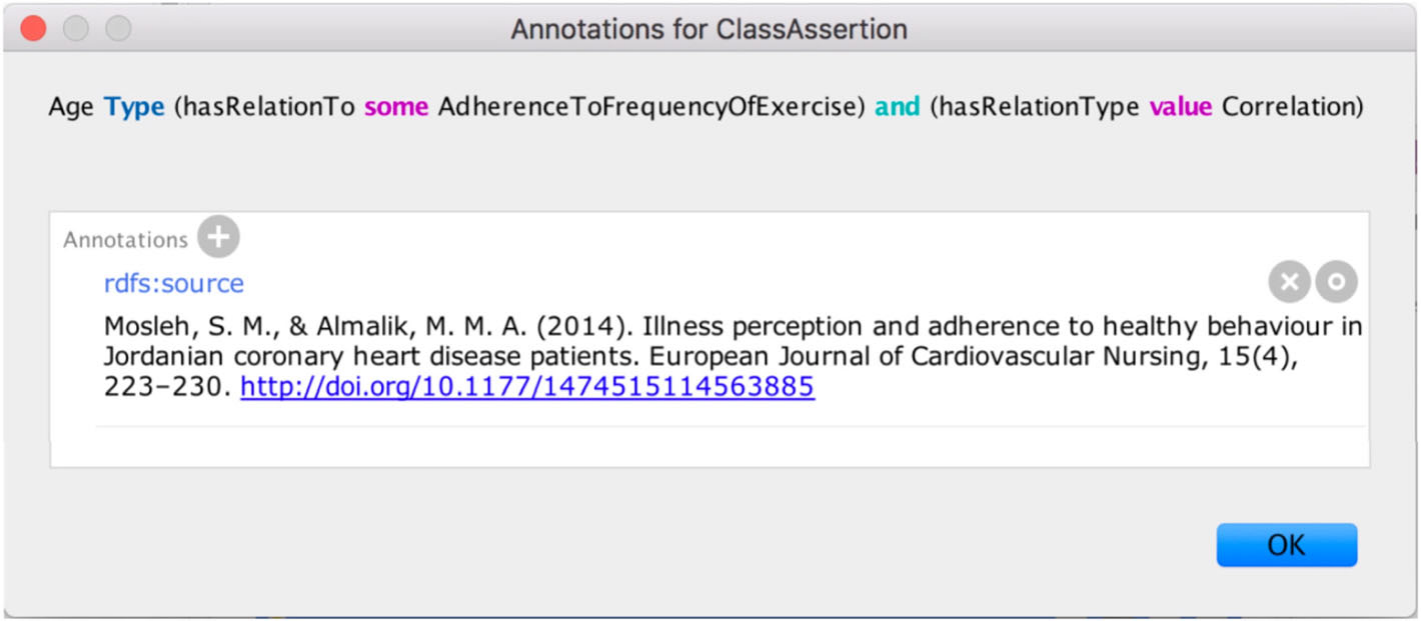
Annotation of the research source in Protégé

Five queries were constructed and returned the results depicted in Fig. 8. Such determinants such as a feeling of no necessity to exercise, feeling too sick or too tired, lack of energy and physical fatigue, and an inconvenient fit of CRP, were yielded as a response to give the reasons for non-adherence to a 1-year-duration cardiac rehabilitation program in women patients (Query 1). The older female factor was found as a patient determinant that facilitates adherence to long-term outpatient cardiac rehabilitation (Query 2). The retrieved results for the factors variously related to adherence in patients with heart disease who have depression are fear of exercise, lack of knowledge about exercise, low mood, low motivation to exercise, negative perception of health, physical restrictions, positive social support, etc. (Query 3). Factors including age, discharge diagnosis, education, ethnicity, exercise habits, perceived control, and other found as predictors of adherence to exercise after index hospitalization (Query 4). Having reason to exercise, perceived psychological benefits of exercise, positive social support, and using psychological strategies are the retrieved facilitators of physical activity adherence after cardiac rehabilitation program in patients with depression (Query 5). The cardiologist stated that the results of Query 1 and Query 2 could be instantly applied in practice of CRP planning and modification of already ongoing CRPs. The output of Query 3 and Query 4 was reviewed as useful for further factor investigation, the results of which might shape the design of CRP or other intervention. The output of Query 5 was evaluated as fair to be employed in the intervention to support patient adherence after CRPs. Additionally, the cardiologist mentioned the advantage of the availability of the evidence-supporting sources of the results. In practice, if the doctor desires to be certain about the evidence that will be considered, the developed ontology promotes such feature through annotations (see Fig. 9).

The three CR trainers who were involved in the evaluation process stated that OPTImAL represents a resource of significant information and is an appropriate support tool in the daily CR practice. One of the trainers proposed that the best fit of the ontology would be when a cardiologist is evaluating a patient’s profile remotely and advises CR trainers regarding maintaining of patient adherence. Regarding ontology usefulness, OPTImAL was evaluated as a valuable tool, combining the physiological factors and the behavioral factors so that it could support the daily practice of trainers and leverage positive outcomes for patients. The importance of the ontology application in clinical practice was highlighted since it facilitates revealing the patients’ factors affecting the participation of CR.

## Discussion

### Outline of achieved objectives

We developed the OPTImAL ontology focusing on physical-activity-related adherence of patients with heart disease. Based on the patient profile, OPTImAL supports the identification of levels and quality of patient adherence to a particular type and dimension of physical activity and exercise training. OPTImAL’s underlying formal model is evidence-based and can serve as a knowledge tool to be used in the practice of a cardiologist or cardiac rehabilitation coach supporting the process of prescription or recommendation of a physical activity regimen. The evaluation of OPTImAL (as described in the section Evaluation of the Results) demonstrated preliminarily that the system could retrieve factors affecting adherence to a plan for a patient with a particular profile. We included specific characteristics of studied populations to support personalization incorporating patient profile. Based on the scientific evidence incorporated in the ontology, the healthcare professional can modify or adjust the action plan promoting patient adherence. From the perspective of knowledge engineering, the ontology may be reused as a part of a broader solution targeting CVD patient adherence through tailoring exercise strategies. In our research, we did not aim to develop a complete system that could be integrated in the healthcare practice without further process of implementation; rather we wanted to approach reusability of evidence-based results related to CVD patients’ activity behavior adherence.

### Interpretation of the results and future work

The heterogeneity of the research results coming from different sources was systematized through the design decisions implemented in the ontology. We centered on expressing the link between three core ideas: (i) adherence, (ii) physical-activity-related behavior, and (iii) affecting factors. To describe these ideas, we specified the concepts that include activity adherence type, quality, and level; activity behavior, stage, dimension and duration; and patient factors, factors relation types, and studied population. Further, we described the relations among these concepts. In total, we characterized relations of 320 factors, which are relevant to different aspects of patient life, to a particular behavior adherence. Factors affecting adherence were categorized by their relevance to the social environment, anthropometric parameters, cognitive states, and other inner and outer patient aspects.

We are aware that semantically, the same information can be represented differently. For this matter, we organized the ontology in a way that it can be extended further concerning its granularity and partially modified respecting the factors thematical classification. The granularity concerns the ability of the model to contain more specific descriptions of the central concepts in the domain of patient adherence and reflect the complexity of the relations among them. By modification of the factors’ thematical classification, we mean the opportunity of the ontology to be updated involving specialists from different related domains (e.g., psychologists, general practitioners, rehabilitation specialist, etc.). So, the grouping of the factors (e.g., patient physiology, demographics, exercise settings, etc.) can be modified according to its domain expert opinion. Further, the interoperability of OPTImAL can be improved by incorporating the ontology medical concept codes from the Unified Medical Language System (UMLS). The purpose of the UMLS concepts is to map different coding systems (e.g., ICD, MeSH, other) to support information exchange between them [35]. Thus, the individual in OPTImAL used as a term for a concept can be labeled with the UMLS concept code. For example, individual PresenceOfCough in OPTImAL can be linked with the UMLS concept [C0010200] Coughing.

We recognize that several concerns should be raised. One of the main concerns we have is rather limited knowledge for constructing such an ontology. Bearing the results of about 50 studies involving CVD patients, we believe that the analysis of available research results should be continued to update the model for its application in medical practice. Also, we didn’t specify synonyms of behaviors, its description, and factors. However, similar expressions were considered as equivalent. For example, factors related to a physical condition being tired such as “FeelingTooTired,” “LackOfEnergy,” and “PhysicalFatigue,” are semantically equivalent in our ontology. We believe that the knowledge base should contain as many synonymous terms as possible to reinforce the (re) usability of the model.

As future work in our ontology development, we aim to elaborate on quantifying the relations among factors. We believe that the practical use of the ontology will increase if the model will be extended with an enumerated estimation of the relations’ effect so that the healthcare expert will appropriately evaluate the importance of the specific factor on the expected patient adherence. For the same matter, the relationships among several adherence determinants may strength the overall estimation of the multiple factors effect on patient adherence. Therefore, the process of expert recommendation or adjustment of the intervention will be more personalized.

## Conclusion

We proposed OPTImAL, a reusable formal model of factors affecting CVD patient adherence to physical activity and exercise. The basis for this model relies on the analysis of data/evidence published in the scientific literature and enables identification of adherence based on the patient profile. In our model, the patient profile is seen from the perspective of 60 multidimensional aspects. We included 320 factors and its relation to patient adherence. Each factor was associated with defined adherence to a particular patient activity behavior. With our model, we are aiming to support the process of a recommendation of physical activity regimen to a patient or to modify the current regimen for better patient adherence. We also see the ontology as a contribution to the development of personalized solutions for physical activity maintenance and modification based on the patient’s need.

OPTImAL was validated for its consistency and positively evaluated by a cardiologist on its appropriateness and usefulness in medical practice. A more formal evaluation study with participation from all intended users of the ontology should be conducted on the further steps of its development.

We believe that our approach is essential in designing decision support systems and strategies for tailored exercise plans or recommendations. Just as decision making in medical practice should be made considering the recent patient condition and medical history, exercise recommendation should be given in connection with a patient profile to support adherence.

## Additional files

Additional file 1: Results of literature analysis: Adherence to physical activity. The file outlines the results of the available published research analysis targeting CVD patient factors related to physical activity adherence. (DOCX 20 kb)

Additional file 2: Results of literature analysis: Adherence to exercise. The file contains an outline of literature analysis focusing on CVD patient factors related to physical exercise. (DOCX 24 kb)

Additional file 3: Results of literature analysis: Adherence to cardiac rehabilitation. The file outlines found in the published research results on CVD patient factors and its relation to cardiac rehabilitation. (DOCX 23 kb)

Additional file 4: Patient factor class hierarchy in Protégé. The file gives a hierarchically-structured list of patient profile classes as organized in Protégé. (DOCX 1451 kb)

Additional file 5: List of the individuals by factors. The file contains a table with all ontology individuals in Protégé. (DOCX 27 kb)

## Abbreviations

CHD: Coronary heart disease
CVD: Cardiovascular disease
OBO: The Open Biological and Biomedical Ontology Foundry
OLS: The Ontology Lookup Service
OWL: Web Ontology Language
WHO: The World Health Organization
