# Supplementary material for: “OPTImAL”: an ontology for patient adherence modeling in physical activity domain
Source: BMC Med Inform Decis Mak. 2019 Apr 25;19:92. doi: 10.1186/s12911-019-0809-9 (PMC6485069; doi:10.1186/s12911-019-0809-9)
Supplement: Supplementary file 1 — Results of literature analysis: Adherence to physical activity. The file outlines the results of the available published research analysis targeting CVD patient factors related to physical activity adherence. (DOCX 20 kb) [file 12911_2019_809_MOESM1_ESM.docx]

Additional file 1. Results of literature analysis: Adherence to physical activity

| Factor | Relation to behavior | Behavior |
| --- | --- | --- |
| Severity of illness (HF patients) [1], self-efficacy (HF patients) [1], goal compatibility (heart failure (HF) patients) [1], timeline (illness perception) [9], personal control (illness perception) [9] | Predictor of adherence | Physical activity |
| Compliance with diet recommendations (HF patients) [1], awareness of disease [10] | Correlation with adherence | Physical activity |
| Higher self-efficacy (HF patients) [1] | Correlation with better adherence | Physical activity |
| NYHA class III (HF patients) [1] | Correlation with worse adherence | Physical activity |
| Family support [5], friends support [5], positive emotional connections with exercise [5] | Motivator | Physical activity |
| Self-efficacy [5], exercise capability [5], value of exercising [5] | Insufficient motivator | Physical activity |
| Non-specific PA instruction [5], fear of negative cardiac consequences of exercise [5], being too busy [5], lack of time to exercise [5], depression [5] | Barrier | Physical activity |
| Coherence (illness perception) [9], body mass index (BMI) [9] | Did not predict | Physical activity |
| Age (HF patients) [1], gender (HF patients) [1], HF diagnosis duration (HF patients) [1], comorbidity (HF patients) [1], education years (HF patients) [1], relationship status (HF patients) [1], work status (HF patients) [1], household income (HF patients) [1] | Not correlated with adherence | Physical activity |
| Physical motivation [6], psychological motivation [6], social motivation [6] | Motivator | Physical activity, level |
| Minor injuries [6], lack of time to exercise [6], family responsibilities [6], physical fatigue [6], physical symptoms [6], working long hours [6], exercise boredom [6], medication side effects [6], inclement weather [6], exercise expenses [6], no interest of exercise in family [6], fear of negative cardiac consequences of exercise [5] | Barrier | Physical activity, level |
| Age [6], marital status [6], New York Heart Association (NYHA) class [6], time after diagnosis [6], smoking [6], alcohol consumption [6], comorbidity [6] | Not associated with adherence | Physical activity, level |
| Higher education [6], higher exercise self-efficacy [6], higher exercise motivation [6] | Association with adherence | Physical activity, high level |
| Inclement weather (HF patients) [4], exercise facility (HF patients) [4], NYHA class (HF patients) [4], social component of exercise (HF patients) [4], relatives encouragement (HF patients) [4], health professionals recommendation (HF patients) [4], self-identity as active (HF patients) [4], doing physical activity in moderation (HF patients) [4], fear of overdoing physical activity (HF patients) [4], personal motivation (HF patients) [4], physical symptoms (HF patients) [4], comorbidity complaints (HF patients) [4], medication (HF patients) [4] | Influencer | Physical activity, regularity |
| Lower perceived susceptibility [2], higher perceived personal control (Arabs only) [2], higher perceived benefit of exercise (only in Jews) [2] | Association with better adherence | Physical activity after index hospitalization |
| Knowledge about disease [7] | Correlation with adherence | Physical activity after cardiac rehabilitation |
| Younger age [3], male [3], cholesterol control [3], fewer comorbidities [3], higher aerobic capacity (VO2) [3], higher perceived health [3], greater PA enjoyment [3], no fear of falling [3], higher household income [3], married [3], full-time work status [3], living outside the city [3], having personal transportation [3], higher social support [3], higher level of perceived healthcare staff support [3] | Correlation with better adherence | Physical activity after cardiac rehabilitation |
| Perceived psychological benefits of exercise (depression population) [8], positive social support (depression population) [8], having reason to exercise (depression population) [8], using psychological strategies (depression population) [8] | Facilitator | Physical activity after cardiac rehabilitation |
| Low mood (depression population) [8], negative perceptions of health (depression population) [8], negative perceptions of life changes (depression population) [8], low motivation to exercise (depression population) [8], perceived external barriers to exercise (depression population) [8], physical restrictions (depression population) [8], fear of exercise (depression population) [8], lack of knowledge about exercise (depression population) [8] | Barrier | Physical activity after cardiac rehabilitation |
| Highest level of education [3], BMI [3], smoking [3], blood pressure control [3], compliance with medication [3], number of recurrent events [3], motivation to exercise [3], ethnicity [3], household income [3], birthplace [3], preferred location of exercise facility [3], number of visits to family doctor [3], number of visits to a cardiologist [3], perceived healthcare staff support [3] | Not correlated with adherence | Physical activity after cardiac rehabilitation |

References

1. Zhang KM, Dindoff K, Arnold JM, Lane J, Swartzman LC. What matters to patients with heart failure? The influence of non-health-related goals on patient adherence to self-care management. Patient Educ Couns. 2015; 98(8):927–34.
2. Reges O, Vilchinsky N, Leibowitz M, Khaskia A, Mosseri M, Kark JD. Illness cognition as a predictor of exercise habits and participation in cardiac prevention and rehabilitation programs after acute coronary syndrome. BMC Public Health. 2013;13:956.
3. Bentley D, Khan S, Oh P, Grace S, Bentley D. Physical activity behavior two to six years following cardiac rehabilitation: a socioecological analysis. Clin Cardiol. 2013;36(2):96–102.
4. Tierney S, Elwers H, Sange C, Mamas M, Rutter MK, Gibson M, Neyses L, Deaton C. What influences physical activity in people with heart failure? A qualitative study. Int J Nurs Stud. 2011;48(10):1234–43.
5. Albert NM, Forney J, Slifcak E, Sorrell J. Understanding physical activity and exercise behaviors in patients with heart failure. Heart Lung. 2015;44(1):2–8.
6. Klompstra L, Jaarsma T, Strömberg A. Physical activity in patients with heart failure: barriers and motivations with special focus on sex differences. Patient Prefer Adherence. 2015;9:1603–10.
7. Lima G, Ghisi DM, Britto R, Motamedi N, Grace SL. Disease-related knowledge in cardiac rehabilitation enrollees: correlates and changes. Patient Educ Couns. 2015;98(4):533–9.
8. Rogerson MC, Murphy BM, Bird S, Morris T. “I don’t have the heart”: a qualitative study of barriers to and facilitators of physical activity for people with coronary heart disease and depressive symptoms. Int J Behav Nutr Phys Act. 2012;9:140.
9. Mosleh SM, Almalik MMA. Illness perception and adherence to healthy behaviour in Jordanian coronary heart disease patients. Eur J Cardiovasc Nurs. 2016;15(4):223–30.
10. Heydari A, Ziaee ES, Gazrani A. Relationship between awareness of disease and adherence to therapeutic regimen among cardiac patients. Int J Community Based Nurs Midwifery. 2015;3(1):23–30.
