# Supplementary material for: “OPTImAL”: an ontology for patient adherence modeling in physical activity domain
Source: BMC Med Inform Decis Mak. 2019 Apr 25;19:92. doi: 10.1186/s12911-019-0809-9 (PMC6485069; doi:10.1186/s12911-019-0809-9)
Supplement: Supplementary file 2 — Results of literature analysis: Adherence to exercise. The file contains an outline of literature analysis focusing on CVD patient factors related to physical exercise. (DOCX 24 kb) [file 12911_2019_809_MOESM2_ESM.docx]

Additional file 2. Results of literature analysis: Adherence to exercise

| Factor | Relation to behavior | Behavior |
| --- | --- | --- |
| Higher mental health (heart failure (HF) patients) [8], higher physical health (HF patients) [8], lower neuroticism (HF patients) [8], health status (HF patients) [16], self-efficacy (HF patients) [16] | Predictor of adherence | Exercise |
| Perceived self-concept as challenge (HF patients) [1], mental health (HF patients) [8], physical health (HF patients) [8], health satisfaction (HF patients) [8], neuroticism (HF patients) [8] | Correlation with adherence | Exercise |
| Perceived self-concept as threat (HF patients) [1] | Correlation with adherence, inverse | Exercise |
| Higher goal compatibility (HF patients) [3] | Correlation with better adherence | Exercise |
| New York Heart Association (NYHA) Class III symptoms [14], NYHA Class IV symptoms [14], worse baseline exercise capacity [14] | Association with poor adherence | Exercise |
| Family support [18], friends support [18], positive emotional connection with exercise [18] | Motivator | Exercise |
| Self-efficacy [18], exercise capability [18], value of exercising [18] | Insufficient motivator | Exercise |
| Lack of self-motivation (HF patients) [8], lack of energy (HF patients) [8], physical symptoms (HF patients) [8], non-specific physical activity instruction [18], fear of negative cardiac consequences of exercise [18], being too busy [18], lack of time to exercise [18], depression [18] | Barrier | Exercise |
| Age [1], sex (HF patients) [1], educational level (HF patients) [1], health insurance (HF patients) [1], occupation (HF patients) [1], length of heart failure diagnosis (HF patients) [1], age (HF patients) [8], race (HF patients) [8], education (HF patients) [8], marital status (HF patients) [8] | Not correlated with adherence | Exercise |
| Younger age (Jordanian patients) [2], lower body mass index (BMI) (Jordanian patients) [2] | Predictor of adherence | Exercise, frequency |
| Consequences, timeline, personal control, coherence, concern (illness perception) [20], age [20], BMI [20] | Correlation with adherence | Exercise, frequency |
| Gender (Jordanian patients) [2], married (Jordanian patients) [2], chronic back pain (Jordanian patients) [2], health education (Jordanian patients) [2] | Did not predict | Exercise, frequency |
| Medication adherence score [20], education [20], treatment control, identity (illness perception) [20] | Not correlated with adherence | Exercise, frequency |
| High social support (HF patients) [9] | Predictor of adherence | Exercise, regularity |
| Depression (patients after myocardial infarction (MI) [15], mood disorder (patients after MI) [15] | Association with adherence, inverse | Exercise, regularity |
| Higher basal score (patients with acute myocardial infarction (AMI) [19], referral to cardiac rehabilitation (patients with AMI) [19], referral to post-discharge cardiac visit (patients with AMI) [19] | Association with good adherence | Exercise, regularity |
| Higher income (patients after AMI) [6] | Association with better adherence | Exercise, regularity |
| Female (patients with AMI) [19], diabetes (patients with AMI) [19], cancer (patients with AMI) [19] | Association with poor adherence | Exercise, regularity |
| Lower education (patients after AMI) [6], anxiety presence (patients after MI) [15] | Association with lower adherence (less adherent) | Exercise, regularity |
| Depression (patients after MI) [15], low social support (HF patients) [9], medium social support (HF patients) [9] | Did not predict | Exercise, regularity |
| Instrumental social support (women) [7] | Predictor of adherence | Exercise after cardiac rehabilitation, persistence |
| Age (women) [7], education (women) [7], BMI (women) [7], NYHA class (women) [7], comorbidity (women) [7], muscle pain (women) [7], joint pain (women) [7], motivation (women) [7], mood states (women) [7], expressive social support (women) [7], self-efficacy (women) [7], benefits (women) [7], barriers (women) [7], exercise experience (women) [7] | Did not predict | Exercise after cardiac rehabilitation, persistence |
| Fear of death [4], ill health avoidance [4], critical incidents [4], independence, becoming a burden, functional fitness, isolation (overcoming aging) [4], social component of exercise [4], being able to enjoy life [4], action planning [4], improved mental health [4] | Influencer | Exercise after cardiac rehabilitation, maintenance (long term) |
| Comorbidity (women) [7] | Predictor of adherence | Exercise after cardiac rehabilitation, intensity |
| Age (women) [7], education (women) [7], BMI (women) [7], NYHA class (women) [7], muscle pain (women) [7], joint pain (women) [7], motivation (women) [7], mood states (women) [7], instrumental social support (women) [7], expressive social support (women) [7], self-efficacy (women) [7], benefits (women) [7], barriers (women) [7], exercise experience (women) [7] | Did not predict | Exercise after cardiac rehabilitation, intensity |
| Benefits (women) [7], barriers (women) [7] | Predictor of adherence | Exercise after cardiac rehabilitation, total amount of exercise |
| Motivational stage [13] | Association with adherence | Exercise after cardiac rehabilitation, total amount of exercise |
| Age (women) [7], education (women) [7], BMI (women) [7], NYHA class (women) [7], comorbidity (women) [7], muscle pain (women) [7], joint pain (women) [7], motivation (women) [7], mood states (women) [7], instrumental social support (women) [7], expressive social support (women) [7], self-efficacy (women) [7], exercise experience (women) [7] | Did not predict | Exercise after cardiac rehabilitation, total amount of exercise |
| Exercise habits (during index hospitalization) [5], ethnicity [5], education [5] | Predictor of adherence | Exercise after index hospitalization |
| Personal control [5] | Association with adherence | Exercise after index hospitalization |
| Perceived susceptibility to heart disease [5] | Association with adherence, inverse | Exercise after index hospitalization |
| Gender [5], age [5], economic situation [5], insurance organization membership [5], discharge diagnosis [5], hospitalization unit [5], history of ischemic heart disease [5] | Did not predict | Exercise after index hospitalization |
| Perceived severity of heart disease [5], perceived benefits of exercise [5] | Not associated with adherence | Exercise after index hospitalization |
| Action planning (patients with uncomplicated MI) [11], self-efficacy recovery (patients with uncomplicated MI) [11], self-motivation [21] | Predictor of adherence | Exercise after cardiac rehabilitation |
| Stages of transtheoretical model [12] | Association with adherence | Exercise after cardiac rehabilitation |
| Married [14], living with partner [14] | Association with better adherence | Exercise after cardiac rehabilitation |
| Younger age [14], female [14], black [14], higher BMI [14], lower quality of life [14], higher levels of depression [14] | Association with poor adherence | Exercise after cardiac rehabilitation |
| Higher negative decisional balance scores [13], lower self-efficacy [13], lack of behavioral change support [13] | Association with lower adherence (less adherent) | Exercise after cardiac rehabilitation |
| Higher education [14], prior heart failure hospitalizations [14], VE/VCO2 slope [14], depression Beck score [14] | Not associated with adherence | Exercise after cardiac rehabilitation |
| Exercise capacity [17], perceived control over heart disease [17] | Predictor of adherence | Exercise during cardiac rehabilitation, frequency |
| Age [17], gender [17], income [17], education [17], social status [17], task self-efficacy [17], coping self-efficacy [17], scheduled self-efficacy [17] | Did not predict | Exercise during cardiac rehabilitation, frequency |

References

1. Heydari A, Ahrari S, Vaghee S. The relationship between self-concept and adherence to therapeutic regimens in patients with heart failure. J Cardiovasc Nurs. 2011;26(6):475–80.
2. Mosleh SM, Darawad M. Patients’ adherence to healthy behavior in coronary heart disease: risk factor management among Jordanian patients. J Cardiovasc Nurs. 2015;30(6):471–8.
3. Zhang KM, Dindoff K, Arnold JM, Lane J, Swartzman LC. What matters to patients with heart failure? The influence of non-health-related goals on patient adherence to self-care management. Patient Educ Couns. 2015; 98(8):927–34.
4. Hardcastle SJ, Mcnamara K, Tritton L. Using visual methods to understand physical activity maintenance following cardiac rehabilitation. PLoS One. 2015;10(9):e0138218
5. Reges O, Vilchinsky N, Leibowitz M, Khaskia A, Mosseri M, Kark JD. Illness cognition as a predictor of exercise habits and participation in cardiac prevention and rehabilitation programs after acute coronary syndrome. BMC Public Health, 2013;13:956.
6. Chan RH, Gordon NF, Chong A. Influence of socioeconomic status on lifestyle behavior modifications among survivors of acute myocardial infarction. Am J Cardiol. 2008;102(12):1583–8.
7. Moore SM, Dolansky MA, Ruland CM, Pashkow FJ, Blackburn GG. Predictors of women’s exercise maintenance after cardiac rehabilitation. J Cardiopulm Rehabil. 2003;23(1):40–9.
8. Evangelista LS, Berg J, Dracup K. Relationship between psychosocial variables and compliance in patients with heart failure. Heart and Lung. 2001;30(4):294–301.
9. Gallagher R, Luttik M, Jaarsma T. Social support and self-care in heart failure. J Cardiovasc Nurs. 2011;26(6):439–445.
10. Blanchard CM, Rodgers WM, Courneya KS, Daub B, Black B. Self-efficacy and mood in cardiac rehabilitation: Should gender be considered? Behav Med. 2002;27(4):149–60.
11. Schwarzer R, Luszczynska A, Ziegelmann JP, Scholz U, Lippke S. Social-cognitive predictors of physical exercise adherence: three longitudinal studies in rehabilitation. Health Psychol. 2008;27(1):54–63.
12. Hellman EA. Use of the stages of change in exercise adherence model among older adults with a cardiac diagnosis. J Cardiopulm Rehabil. 1997;17(3):145–55.
13. Bock BC, Albrecht AE, Traficante RM, Clark MM, Pinto BM, Tilkemeier P, Marcus BH. Predictors of exercise adherence following participation in a cardiac rehabilitation program. Int J Behav Med. 1997;4(1):60–75.
14. Cooper LB, Mentz RJ, Sun J, Schulte PJ, Fleg JL, Cooper LS, Connor CM. Psychosocial factors, exercise adherence, and outcomes in heart failure patients: insights from heart failure: a controlled trial investigating outcomes of exercise training (HF-ACTION). Circ Heart Fail. 2015;8(6):1044–51.
15. Kuhl EA, Fauerbach JA, Bush DE, Ziegelstein RC. Relation of anxiety and adherence to risk-reducing recommendations following myocardial infarction. Americal J Cardiol. 2009;103(12):1629–34.
16. Subramanian U, Hopp F, Mitchinson A, Lowery J. Impact of provider self-management education, patient self-efficacy, and health status on patient adherence in heart failure in a Veterans Administration population. Congest Heart Fail. 2008;14(1):6–11.
17. Murray T, Rodgers W. The role of socioeconomic status and control beliefs on frequency of exercise during and after. Appl Psychol Health Well Being. 2012;4(1):49–66.
18. Albert NM, Forney J, Slifcak E, Sorrell J. Understanding physical activity and exercise behaviors in patients with heart failure. Heart Lung. 2015;44(1):2–8.
19. Urbinati S, Olivari Z, Gonzini L, Savonitto S, Farina R, Del Pinto M, Valbusa A, Fantini G, Mazzoni A, Maggioni AP; BLITZ-4 Investigators. Secondary prevention after acute myocardial infarction: drug adherence, treatment goals, and predictors of health lifestyle habits. The BLITZ-4 Registry. Eur J Prev Cardiol. 2015;22(12):1548–56.
20. Mosleh SM, Almalik MM. Illness perception and adherence to healthy behaviour in Jordanian coronary heart disease patients. Eur J Cardiovasc Nurs. 2014;15(4):223–30.
21. Russell KL, Bray SR. Self-determined motivation predicts independent, home-based exercise following cardiac rehabilitation. Rehabil Psychol. 2009;54(2):150–6.
