# Supplementary material for: “OPTImAL”: an ontology for patient adherence modeling in physical activity domain
Source: BMC Med Inform Decis Mak. 2019 Apr 25;19:92. doi: 10.1186/s12911-019-0809-9 (PMC6485069; doi:10.1186/s12911-019-0809-9)
Supplement: Supplementary file 3 — Results of literature analysis: Adherence to cardiac rehabilitation. The file outlines found in the published research results on CVD patient factors and its relation to cardiac rehabilitation. (DOCX 23 kb) [file 12911_2019_809_MOESM3_ESM.docx]

Additional file 3. Results of literature analysis: Adherence to cardiac rehabilitation

| Factor | Relation to cardiac rehabilitation | Cardiac rehabilitation program duration |
| --- | --- | --- |
| Irrational health beliefs [16] | Predictor of CR adherence | Up to 3 months |
| Older age [16], higher income [16] | Positive association with cardiac rehabilitation | Up to 3 months |
| Physician recommendation (women) [18], health professional recommendation (women) [18], feeling of necessity of exercise for recovery (women) [18], reminder of cardiac rehabilitation program (CRP) (women) [18], benefits of cardiac rehabilitation (CR) attendance in others (women) [18] | Reasons for CR attendance | Up to 3 months |
| Myocardial infarction (MI) diagnosis (women) [18], unemployed (women) [18], retired (women) [18], home stress (women) [18], perceived control (women) [18] | Predictor of CR nonadherence | Up to 3 months |
| Younger age [11], being employed [11] | Positive association with CR noncompleting | Up to 3 months |
| Physical unfitness [8], work conflicts [8], time conflicts [8], scheduled cardiac interventions [8], lack of referrals to CR [8,18 (women)], noncompliance with cardiac treatment [8], financial constraints [8], self-exercise [8], fear after exercise stress testing [8], treatment [8], feeling of necessity of CR exercise [8], received CR services before [8], pending non-cardiac surgery [8], no support from family [8], living far from CR facility [8] | Barrier to CR attendance | Up to 3 months |
| Transportation problems (women) [18], feeling too sick (women) [18], feeling too tired (women) [18], feeling of no necessity of CR exercise (women) [18], lack of motivation (women) [18], inconvenient fit of CRP (women) [18], not recommended as necessary by doctor (women) [18] | Reasons for CR nonadherence | Up to 3 months |
| Lack of interest [5], language-related issues in CR [5] | Reason for refusing CR participation | Up to 3 months |
| Antidepressant medication (patients after cardiac surgery) [12], overweight (patients after cardiac surgery) [12], being single (patients after cardiac surgery) [12], post-discharge readmission (patients after cardiac surgery) [12] | Predictor of CR dropout | Up to 3 months |
| Lower behavioral intentions (patients with uncomplicated MI) [6], lower action self-efficacy (patients with uncomplicated MI) [6] | Positive association with CR dropout | Up to 3 months |
| Retreatment [11], new surgical procedure [11], intensive treatment [11], work conflicts [11], family responsibilities [11] | Reason for CR dropout | Up to 3 months |
| Low exercise capacity [14], high body mass index (BMI) [14], smoking [14], diabetes [14], widowhood [14] | Predictor of early dropout | Up to 3 months |
| Referral for cardiac surgery [14], referral to inpatient program [14], orthopedic issues [14], psychological issues [14], vascular issues [14] | Reason for early dropout | Up to 3 months |
| Gender (patients with uncomplicated MI) [6], age (patients with uncomplicated MI) [6] | Not significant association with CR adherence | Up to 3 months |
| Age (patients after cardiac surgery) [12], gender [16], gender (patients after cardiac surgery) [12], smoking (patients after cardiac surgery) [12], type of surgery (patients after cardiac surgery) [12], educational attainment [16], marital status [16], employment status [16], The Center for Epidemiologic Studies Depression Scale (CES-D) subscales (somatic, emotional well-being, depressive affect, interpersonal) [16] | Not related to CR adherence | Up to 3 months |
| Gender [11], ethnicity [11], education [11], marital status [11], reason for referral [11], depression [16] | Not related to CR completing | Up to 3 months |
| Depression [9], BMI [9], dysphoric mood [9] | Predictor of CR adherence | Up to 1 year |
| Diabetes [4], obesity [4] | Predictor of CR nonadherence | Up to 1 year |
| South Asians [7] | Positive association with CR noncompleting | Up to 1 year |
| Higher depression score [17], male [17], less social support [17] | Predictor of CR dropout | Up to 1 year |
| Higher levels of depression [9], neuroticism [9], physical symptoms [9], lower level of optimism [9], diabetes (heart transplant recipients) [15], abdominal obesity (heart transplant recipients) [15], lower peak diastolic blood pressure (heart transplant recipients) [15] | Positive association with CR dropout | Up to 1 year |
| Health problems [9,15 (heart transplant recipients),17], work conflicts [9], problems with insurance [9], change of domicile [9,17], loss of interest [9], lack of interest [17], musculoskeletal issues [17], vascular issues [17], angioplasty [17], transportation problems [17], employment issues [17], psychological issues [17], language-related issue in CR [17], diagnosis of brain tumor [17], hip replacement surgery [17], coronary artery bypass surgery [17], musculoskeletal issue [17], employment issues [17] | Reason for CR dropout | Up to 1 year |
| Age [17], sex [17], BMI [17], peak oxygen uptake (VO2peak) [17], 6-minute walk distance (6MWD) [17], CESD score (Center for epidemiologic studies depression scale) [17] | Not related to CR adherence | Up to 1 year |
| Age [9], oxygen consumption (VO2 max) [9], BMI [9], left ventricular ejection fraction (LVEF) [9] | Not related to CR completing | Up to 1 year |
| Age [2], high-density lipoprotein cholesterol (HDL-C) [2], exercise test peak heart rate [2], last completed stage [2] | Positive association with CR adherence | Up to 3 years |
| BMI [2], sum of three skinfolds [2], total cholesterol [2], triglycerides [2] | Inverse association with CR adherence | Up to 3 years |
| Smoking [2] | Barrier to CR attendance | Up to 3 years |
| Smoking [1], blue collar occupation [1], presence of cough [1], younger age [1], higher systolic blood pressure [1], history of angina [1] | Positive association with CR dropout | Up to 3 years |
| Change of job [1], change of domicile [1], inconvenient time for CR exercise [1], being too busy [1], psychosocial lack of motivation [1], lack of interest [1], angina [1], arthritis [1], alcoholism [1] | Reason for CR dropout | Up to 3 years |
| Cholesterol [1], triglyceride [1], ponderal index [1], depression score [2] | Not significant association with CR adherence | Up to 3 years |
| Exercise intensity [1], low-density lipoprotein cholesterol (LDL-C) [2], educational status [2], peak exercise systolic blood pressure [2], number of previous myocardial infarctions [2], time since last infarction [2], alcohol consumption [2], resting systolic blood pressure [2] | Not related to CR adherence | Up to 3 years |
| Older female [10] | Facilitator of CR attendance | Longer than 3 years |
| Younger female [10], smoking [10], home stress (men) [10], presence of a family history of premature coronary disease (men) [10] | Barrier to CR attendance | Longer than 3 years |
| Angina [10], coronary angioplasty [10], myocardial infarction [10], coronary artery bypass surgery [10], pacemaker implantation [10], transportation problems [10], work conflicts [10] | Reasons for CR nonadherence | Longer than 3 years |
| Age [10] | Not related to CR completing | Longer than 3 years |
| Perceived benefits of regular exercise [3], personal control [3], ethnicity [3], discharge diagnosis [3], history of ischemic heart disease [3] | Predictor of CR adherence | Not defined |
| Travel [13], work responsibilities [13], inclement weather [13], time constraints [13], health problems [13], feeling pain from exercise [13], lack of energy [13], family responsibilities [13], preference to exercise alone [13], long waiting for CR referral [13], feeling of no necessity of CR exercise [13],not recommended as necessary by doctor [13], feeling too old [13], no contact from CR staff [13], not knowing about CR [13], Already exercise at home or in community [13], inconvenient location of exercise facility [13], CR expenses [13], transportation problems [13] | Barrier to CR attendance | Not defined |
| Age [3], subjective socioeconomic position [3], marital status [3], education [3], insurance organization membership [3] | Not significant association with CR adherence | Not defined |

References

1. Oldridge NB, Donner AP, Buck CW, Jones NL, Andrew GM, Parker JO, Cunningham DA, Kavanagh T, Rechnitzer PA, Sutton JR. Predictors of dropout from cardiac exercise rehabilitation. Am J Cardiol. 1983;51:70–4.
2. Dorn J, Naughton J, Imamura DA, Trevisan M. Correlates of compliance in a randomized exercise trial in myocardial infarction patients. Med Sci Sports Exerc. 2001;33(7):1081–9.
3. Reges O, Vilchinsky N, Leibowitz M, Khaskia A, Mosseri M, Kark JD. Illness cognition as a predictor of exercise habits and participation in cardiac prevention and rehabilitation programs after acute coronary syndrome. BMC Public Health. 2013;13:956.
4. Forhan M, Zagorski BM, Marzonlini S, Oh P, Alter DA. Predicting exercise adherence for patients with obesity and diabetes referred to a cardiac rehabilitation and secondary prevention program. Can J Diabetes, 2013;37(3):189–94.
5. Blanchard CM, Rodgers WM, Courneya KS, Daub B, Black B. Self-efficacy and mood in cardiac rehabilitation: should gender be considered? Behav Med. 2002;27(4):149–60.
6. Schwarzer R, Luszczynska A, Ziegelmann JP, Scholz U, Lippke S. Social-cognitive predictors of physical exercise adherence: three longitudinal studies in rehabilitation. Health Psychol. 2008;27(1):54–63.
7. Banerjee AT, Gupta M, Singh N. Patient Characteristics, compliance, and exercise outcomes of South Asians enrolled in cardiac rehabilitation. J Cardiopulm Rehabil. 2007;27:212–8.
8. Mak Y, Chan W, Yue C. Barriers to participation in a phase II cardiac rehabilitation programme. Hong Kong Med J. 2005;11(6):472–5.
9. Glazer KM, Emery CF, Frid DJ, Banyasz RE. Psychological predictors of adherence and outcomes among patients in cardiac rehabilitation. J Cardiopulm Rehabil. 2002;22(1):40–6.
10. Cannistra LB, Balady GJ, O'Malley CJ, Weiner DA, Ryan TJ. Comparison of the clinical profile and outcome of women and men in cardiac rehabilitation. Am J Cardiol. 1992;69(16):1274–9.
11. Bock, B. C., Albrecht, A. E., Traficante, R. M., Clark, M. M., Pinto, B. M., Tilkemeier, P., & Marcus, B. H. (1997). Predictors of exercise adherence following participation in a cardiac rehabilitation program. International Journal of Behavioral Medicine, 4(1), 60–75.
12. Laustsen S, Hjortdal VE, Petersen AK. Predictors for not completing exercise-based rehabilitation following cardiac surgery following cardiac surgery. Scand Cardiovasc J. 2013;47:344–51.
13. Shanmugasegaram S, Oh P, Reid RD, McCumber T, Grace SL. A comparison of barriers to use of home versus site-based cardiac rehabilitation. J Cardiopulm Rehabil. 2015;33(5):297–302.
14. Wittmer M, Volpatti M, Piazzalonga S, Hoffmann A. Expectation, satisfaction, and predictors of dropout in cardiac rehabilitation. European Journal of Preventive Cardiology, 2011;19(5):1082–8.
15. Marzolini S, Grace SL, Brooks D, Corbett D, Mathur S, Bertelink R, Oh P. Time-to-referral, use, and efficacy of cardiac rehabilitation after heart transplantation. Transplantation. 2015;99(3):594–601.
16. Anderson DR, & Emery CF. Irrational health beliefs predict adherence to cardiac rehabilitation: a pilot study. Health Psychol. 2014;33(12):1614–7.
17. Marzolini S, Danells C, Oh PI, Jagroop D, Brooks D. Feasibility and effects of cardiac rehabilitation for individuals after transient ischemic attack. J Stroke Cerebrovasc Dis. 2016;25(10):2453–63.
18. Gallagher R, McKinley S, Dracup K. Predictors of women’s attendance at cardiac rehabilitation programs. Progr Cardiovasc Nurs. 2003;18(3):121–6.
