# Supplementary figures and images for: “OPTImAL”: an ontology for patient adherence modeling in physical activity domain
Source: BMC Med Inform Decis Mak. 2019 Apr 25;19:92. doi: 10.1186/s12911-019-0809-9 (PMC6485069; doi:10.1186/s12911-019-0809-9)

Additional file 4. Patient factor class hierarchy in Protégé


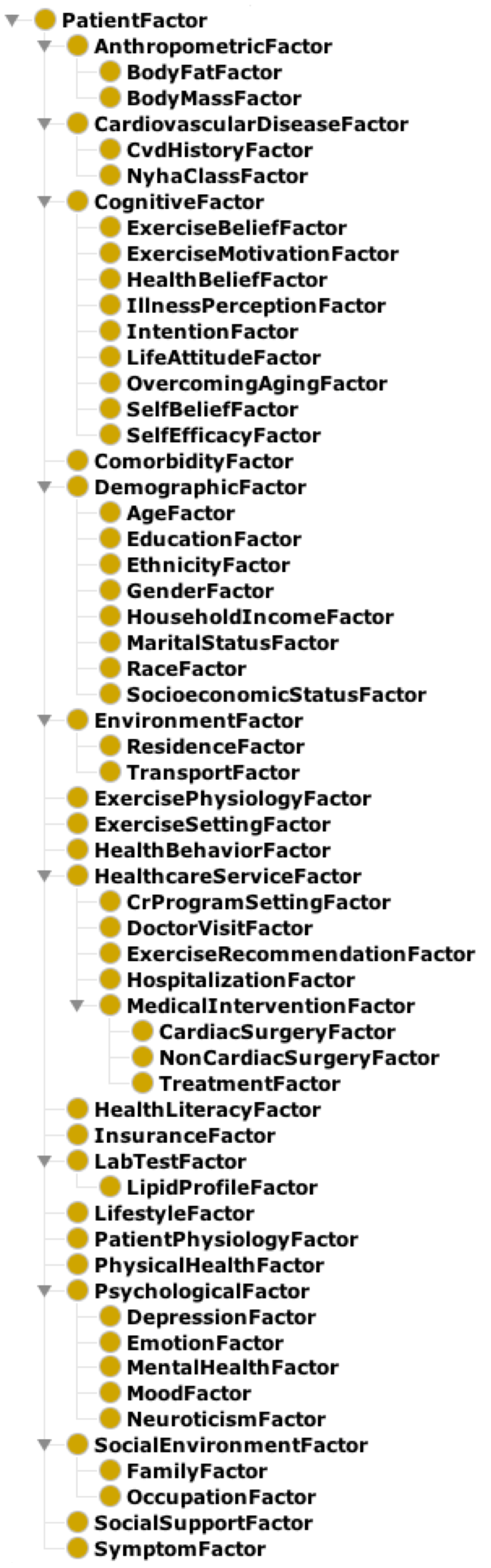

Supplement: Supplementary file 4 — Patient factor class hierarchy in Protégé. The file gives a hierarchically-structured list of patient profile classes as organized in Protégé. (DOCX 1451 kb) [file 12911_2019_809_MOESM4_ESM.docx]
