# Supplementary material for: “OPTImAL”: an ontology for patient adherence modeling in physical activity domain
Source: BMC Med Inform Decis Mak. 2019 Apr 25;19:92. doi: 10.1186/s12911-019-0809-9 (PMC6485069; doi:10.1186/s12911-019-0809-9)
Supplement: Supplementary file 5 — List of the individuals by factors. The file contains a table with all ontology individuals in Protégé. (DOCX 27 kb) [file 12911_2019_809_MOESM5_ESM.docx]

Additional file 5. List of the individuals by factors

| ClassFactor | Individuals |
| --- | --- |
| Body Fat Factor  (Anthropometric Factor) | SumOfThreeSkinfolds |
| Body Mass Factor  (Anthropometric Factor) | PonderalIndex  HigherBodyMassIndex  HighBodyMassIndex  BodyMassIndex  Overweight  LowerBodyMassIndex |
| CVD History Factor  (Cardiovascular Disease Factor) | CriticalIncidents  DischargeDiagnosis  HeartFailureDiagnosisDuration  HistoryOfIschemicHeartDisease  LengthOfHeartFailureDiagnosis  MyocardialInfarctionDiagnosis  NumberOfPreviousMyocardialInfarctions  NumberOfRecurrentEvents  PostDischargeReadmission  PriorHeartFailureHospitalizations  SeverityOfIllness  TimeAfterDiagnosis  TimeSinceLastInfarction |
| NYNA Factor  (Cardiovascular Disease Factor) | NyhaClass  NyhaClass3  NyhaClass4  PhysicalRestrictions |
| Exercise Belief Factor  (Cognitive Factor) | Barriers  Benefits  BenefitsOfCrAttendanceInOthers  FeelingOfNecessityOfExerciseForRecovery  FeelingOfNoNecessityOfCrExercise  HigherPerceivedBenefitOfExercise  PerceivedBenefitsOfExercise  PerceivedBenefitsOfRegularExercise  PerceivedExternalBarriersToExercise  PerceivedPsychologicalBenefitsOfExercise  ValueOfExercise |
| Exercise Motivation Factor  (Cognitive Factor) | HavingReasonToExercise  HigherExerciseMotivation  LackOfMotivation  LackOfSelfMotivation  LowMotivationToExercise  Motivation  MotivationalStage  MotivationToExercise  PersonalMotivation  PhysicalMotivation  PsychologicalMotivation  PsychosocialLackOfMotivation  SelfMotivation  SocialMotivation |
| Health Belief Factor  (Cognitive Factor) | HealthSatisfaction  HigherPerceivedHealth  IrrationalHealthBeliefs  LowerPerceivedSusceptibility  NegativePerceptionOfHealth  PerceivedSeverityOfHeartDisease  PerceivedSusceptibilityToHeartDisease |
| Illness Perception Factor  (Cognitive Factor) | Coherence  Concern  Consequences  HigherPerceivedPersonalControl  Identity  PerceivedControl  PerceivedControlOverHeartDisease  PersonalControl  SelfIdentityAsActive  Timeline  TreatmentControl |
| Intention Factor  (Cognitive Factor) | ActionPlanning  GoalCompatibility  HigherGoalCompatibility  HigherNegativeDecisionalBalanceScore  LowerBehavioralIntentions  StagesOfTranstheoreticalModel  UsingPsychologicalStrategies |
| Life Attitude Factor  (Cognitive Factor) | BeingAbleToEnjoyLife  LowerLevelOfOptimism  LowerQualityOfLife  NegativePerceptionsOfLifeChanges |
| Overcoming Aging Factor  (Cognitive Factor) | BecomingBurden  FeelingTooOld  FunctionalFitness  Independence  Isolation |
| SelfBelief Factor  (Cognitive Factor) | PerceivedSelfConceptAsChallange  PerceivedSelfConceptAsThreat |
| SelfEfficacy Factor  (Cognitive Factor) | HigherExerciseSelfEfficacy  HigherSelfEfficacy  LowerActionSelfEfficacy  LowerSelfEfficacy  SelfEfficacy  SelfEfficacyCoping  SelfEfficacyRecovery  SelfEfficacyScheduling  SelfEfficacyTask |
| Comorbidity Factor | AbdominalObesity  Alcoholism  Arthritis  Cancer  Comorbidity  Diabetes  DiagnosisOfBrainTumor  FewerComorbidities  Obesity |
| Age Factor  (Demographic Factor) | Age  OlderAge  YoungerAge |
| Education Factor  (Demographic Factor) | Education  EducationalAttainment  EducationalLevel  EducationalStatus  EducationYears  HigherEducation  HighestLevelOfEducaiton  LowerEducation |
| Ethnicity Factor  (Demographic Factor) | Ethnicity |
| Gender Factor  (Demographic Factor) | Female  Gender  Male  OlderFemale  YoungerFemale |
| Household Income Factor  (Demographic Factor) | FinancialConstraints  HigherHouseholdIncome  HigherIncome  HouseholdIncome  Income |
| Marital Status Factor  (Demographic Factor) | BeingSingle  LivingWithPartner  MaritalStatus  Married  RelationshipStatus  Widowhood |
| Race Factor  (Demographic Factor) | Black  Race  SouthAsians |
| Socioeconomic Status Factor  (Demographic Factor) | EconomicSituation  SocialStatus  SubjectiveSocioeconomicPosition |
| Residence Factor  (Environment Factor) | Birthplace  ChangeOfDomicile  InclementWeather  LivingOutsideTheCity |
| Transport Factor  (Environment Factor) | HavingPersonalTransportation  TransportationProblems |
| Exercise Physiology Factor | 6MinuteWalkDistance  ExerciseCapability  ExerciseCapacity  ExerciseTestPeakHeartRate  HigherAerobicCapacity  HigherBasalScore  LowExerciseCapacity  OxygenConsumption  PeakExerciseSystolicBloodPressure  PeakOxygenUptake  VeVco2Slope  WorseBaselineExerciseCapacity |
| Exercise Setting Factor | ExerciseExpenses  ExerciseFacility  ExerciseIntensity  InconvenientLocationOfExerciseFacility  PreferenceToExerciseAlone  PreferredLocationOfExerciseFacility  SocialComponentOfExercise |
| Health Behavior Factor | BloodPressureControl  CholesterolControl  ComplianceWithDietRecommendations  ComplianceWithMedication  DoingPhysicalActivityInModeration  ExerciseExperience  ExerciseHabits  MedicationAdherenceScore  NonComplianceWithCardiacTreatment  SelfExercise |
| CR Program Setting Factor  (subclass of Healthcare Service Factor) | CrExpenses  InconvenientFitOfCrProgram  InconvenientTimeForCrExercise  LackOfReferralsToCr  LanguageRelatedIssueInCr  LastCompletedStage  LivingFarFromCrFacility  LongWaitingForCrReferral  NoContactFromCrStaff  NotKnowingAboutCr  ReasonForReferral  ReceivedCrServicesBefore  ReferralToCr  ReferralToInpatientProgramme  ReminderOfCrProgram |
| Doctor Visit Factor  (Healthcare Service Factor) | NumberOfVisitsToCardiologist  NumberOfVisitsToFamilyDoctor  ReferralToPostDischargeCardiacVisit |
| Exercise Recommendation Factor  (Healthcare Service Factor) | HealthProfessionalRecommendation  NonSpecificPhysicalActivityInstruction  NotRecommendedAsNecessaryByDoctor  PhysicianRecommendation |
| Hospitalization Factor  (Healthcare Service Factor) | HospitalizationUnit |
| Medical Intervention Factor  (Healthcare Service Factor) |  |
| Cardiac Surgery Factor  (Medical Intervention Factor) | Angioplasty  CoronaryAngioplasty  CoronaryArteryBypassSurgery  NewSurgicalProcedure  PacemakerImplantation  ReferralForCardiacSurgery  ScheduledCardiacIntervention  TypeOfSurgery |
| NonCardiac Surgery Factor  (Medical Intervention Factor) | HipReplacementSurgery  PendingNonCardiacSurgery |
| Treatment Factor  (Medical Intervention Factor) | AntidepressantMedication  IntensiveTreatment  Medication  MedicationSideEffects  Retreatment  Treatment |
| Health Literacy Factor | AwarenessOfDisease  HealthEducation  KnowledgeAboutDisease  LackOfKnowledgeAboutExercise |
| Insurance Factor | HealthInsurance  InsuranceOrganizationMembership  ProblemsWithInsurance |
| Lipid Profile Factor  (Lab Test Factor) | Cholesterol  HighDensityLipoproteinCholesterol  LowDensityLipoproteinCholesterol  TotalCholesterol  Triglycerides |
| Lifestyle Factor | AlcoholConsumption  BeingTooBusy  LackOfTimeToExercise  Smoking  TimeConflicts  TimeConstrains  Traveling |
| Patient Physiology Factor | LeftVentricularEjectionFraction  LowerPeakDiastolicBloodPressure  RestingSystolicBloodPressure |
| Physical Health Factor | HealthProblem  HealthStatus  HigherPhysicalHealth  PhysicalHealth  PhysicalUnfitness |
| Depression Factor  (Psychological Factor) | CesdScore  CesdSubscales  Depression  DepressionBeckScore  DepressionScore  HigherDepressionScore  HigherLevelsOfDepression |
| Emotion Factor  (Psychological Factor) | AnxietyPresence  ExerciseBoredom  FearAfterExerciseStressTesting  FearOfDeath  FearOfExercise  FearOfNegativeCardiacConsequencesOfExercise  FearOfOverdoingPhysicalActivity  GreaterPhysicalActivityEnjoyment  LackOfInterest  LossOfInterest  NoFearOfFalling  PositiveEmotionalConnectionWithExercise |
| Mental Health Factor  (Psychological Factor) | HigherMentalHealth  IllHealthAvoidance  ImprovedMentalHealth  MentalHealth  PsychologicalIssues |
| Mood Factor  (Psychological Factor) | DysphoricMood  LowMood  MoodDisorder  MoodStates |
| Neuroticism Factor  (Psychological Factor) | LowerNeuroticism  Neuroticism |
| Family Factor  (Social Environment Factor) | FamilyHistoryOfPrematureCoronaryDisease  FamilyResponsibilities  HomeStress  NoInterestOfExerciseInFamily |
| Occupation Factor  (Social Environment Factor) | BeingEmployed  BlueCollarOccupation  ChangeOfJob  EmploymentIssues  EmploymentStatus  FullTimeWorkStatus  Occupation  Retired  Unemployed  WorkConflicts  WorkingLongHours  WorkResponsibilities  WorkStatus |
| Social Support Factor | ExpressiveSocialSupport  FamilySupport  FriendsSupport  HealthcareStaffSupport  HigherLevelOf PerceivedHealthcareStaffSupport  HigherSocialSupport  HighSocialSupport  InstrumentalSocialSupport  LackOfBehavioralChangeSupport  LessSocialSupport  LowSocialSupport  MediumSocialSupport  NoSupportFromFamily  PerceivedHealthcareStaffSupport  PositiveSocialSupport  RelativesEncouragement |
| Symptom Factor | Angina  ChronicBackPain  ComorbidityComplaints  FeelingPainFromExercise  FeelingTiredFromExercise  FeelingTooSick  FeelingTooTired  HigherSystolicBloodPressure  HistoryOfAngina  JointPain  LackOfEnergy  MinorInjuries  MusclePain  MusculoskeletalIssues  OrthopaedicIssues  PhysicalFatigue  PhysicalSymptoms  PresenceOfCough  VasularIssues |
